# Association between TNFa rs1800629 and migraine: Case-control study and updated meta-analysis

**DOI:** 10.3934/Neuroscience.2026013

**Published:** 2026-05-29

**Authors:** Ioannis Liampas, Maria Papasavva, Silvia Demiri, Michail Vikelis, Chrysoula Marogianni, Evripidis Vlachakis, Emmanouil V Dermitzakis, Aristidis Tsatsakis, Nikolaos Drakoulis, Efthimios Dardiotis, Vasileios Siokas

**Affiliations:** 1 Department of Neurology, University Hospital of Larissa, Faculty of Medicine, University of Thessaly, Mezourlo Hill, 41110 Larissa, Greece; 2 Laboratory of Neurogenetics, University of Thessaly, Mezourlo Hill, 41110 Larissa, Greece; 3 Department of Pharmacy, School of Health Sciences, Frederick University, Nicosia, Cyprus; 4 Research Group of Clinical Pharmacology and Pharmacogenomics, Faculty of Pharmacy, School of Health Sciences, National and Kapodistrian University of Athens, Panepistimiopolis Zografou, 15771, Athens, Greece; 5 School of Medicine, University of Patras, Rio Patras 26504, Greece; 6 Headache Clinic, Mediterraneo Hospital, 166 75 Glifada, Greece; 7 Euromedica General Clinic, 546 45 Thessaloniki, Greece; 8 Center of Toxicology & Science Applications, Medical School, University of Crete, 71003 Heraklion, Greece; 9 Universidad Ecotec, Km. 13.5 Samborondon, EC 092302 Samborondon, Ecuador; 10 Sechenov IM, First State Medical University, 119991 Moscow, Russia

**Keywords:** migraine, rs1800629, TNFa, SNP, polymorphism

## Abstract

Migraine is a multifactorial disorder influenced by both genetic and environmental factors. In this study, we aimed to explore the association of tumor necrosis factor alpha (TNFa) rs1800629 with migraine susceptibility. A case-control study design was employed to assess this association in individuals of Greek ancestry. Subsequently, a meta-analysis of published studies was conducted, thereby incorporating the findings of the present study to further evaluate the relationship between rs1800629 and a migraine. A total of 123 patients with migraines (44.4 ± 10.3 years, 105 women) and an even number of healthy controls (HC) (58.7 ± 12.0 years, 76 women) were recruited. TNFa rs1800629 was in Hardy–Weinberg equilibrium among the HC (P = 1.00). No association was observed between TNFa rs1800629 and migraines under any genetic model. Additional, subgroup analyses stratified by sex and migraine subtype showed no associations. The meta-analysis, comprised of 14,742 participants with migraines and 46,384 HC, indicated a trend towards a risk-conferring effect of rs1800629 [Odds ratio (OR) = 1.26, 95% confidence interval (95% CI) = (0.97–1.64), P = 0.09]. Subgroup analyses revealed a significant association in individuals of Asian ancestry [OR = 1.64, 95% CI = (1.08–2.48), P = 0.02]. Additionally, the over-dominant model was related to migraines with aura [OR = 1.21, 95% CI = (1.08–1.35), P = 0.001]. Subgroup analyses for men and women, as well as for migraines without aura, were insignificant. This case-control study provides evidence that the TNFa rs1800629 polymorphism is not associated with migraine susceptibility in the Greek population. The updated meta-analysis showed that rs1800629 increases migraine risk in individuals of Asian ancestry. These findings support a population-specific genetic effect. Finally, the observed association with migraine with aura under the over-dominant model may indicate a heterozygote-driven effect. Given the extremely low frequency of homozygosity for the minor allele, this result should be interpreted with caution.

## 1. Introduction

Migraine is a complex neurovascular disorder influenced by multiple genetic and environmental factors [1]–[3]. Data from epidemiological studies reveal that the burden of a migraine has increased over recent years, thereby affecting more than one billion individuals worldwide—approximately 14%–15% of the total population—and up to 25% of the employees [4],[5]. Recent genome-wide association studies have identified numerous risk loci, thus advancing our understanding of the highly polygenic nature of migraine [6],[7]. Nevertheless, the underlying pathophysiology remains incompletely understood, with prevailing hypotheses implicating neurovascular and inflammatory mechanisms [8],[9]. The latter involve the release of pro-inflammatory cytokines, including tumor necrosis factor-alpha (TNFa), an effector of immune responses that contributes to neurogenic inflammation and the activation of the trigeminovascular system [10].

The TNFa gene is mapped on chromosome 6p21.33 within the major histocompatibility complex class III region [11]. Numerous single nucleotide polymorphisms (SNPs) within the gene and its promoter region have been linked to a range of autoimmune and chronic inflammatory disorders [12],[13]. Among these SNPs, the TNFa-308 G>A polymorphism (rs1800629), which located in the promoter region, influences transcriptional activity and has been associated with increased TNFa expression in carriers of the A allele [14],[15]. This variant has been linked to susceptibility to several immune-mediated and inflammatory conditions, including rheumatoid arthritis, systemic lupus erythematosus, and inflammatory bowel disease [12],[16]. In this context, several studies have explored the association of TNFa-308 G>A with (1) migraine susceptibility reporting significant associations in Asian populations, as well as with (2) a poorer response to non-steroidal anti-inflammatory drugs [17]–[19].

The present study aimed to explore the potential association of rs1800629 and migraines in individuals of Greek ancestry, using a case-control design. Additionally, we sought to re-evaluate the overall association between rs1800629 and migraines through a meta-analysis of published evidence, with a particular focus on potential sex, ethnic, and migraine subtype differences.

## 2. Materials and methods

### 2.1. Ethical approval and consent to participate

The Ethics Committee of the University of Thessaly and the Mediterraneo Hospital, Glyfada, Greece, approved the case-control study protocol prior to initiation. Written informed consent was obtained from all participants prior to participation.

### 2.2. Case-control study

#### 2.2.1. Participants and settings

Participants with migraines were prospectively enrolled from headache clinics at the General University Hospital of Larissa, in Glyfada and in Thessaloniki, Greece. Migraine diagnoses were established by consultant neurologists with a specific interest in headache medicine, according to the International Classification of Headache Disorders–3rd edition (ICHD-3). Consecutive invitations were extended to all eligible individuals of Greek ancestry, aged over 18 years. Participants with migraines were excluded if they had a prior or concurrent diagnosis of other headache disorder and/or other neurological disease.

Healthy controls (HC) originated from the same community as the cases. The control group consisted of Caucasian adults (>18 years old) of Greek ancestry. HC were excluded if they had a prior or concurrent diagnosis of other headache disorders and/or other neurological diseases. Moreover, those with a family history of migraine were excluded. To establish eligibility, HC were screened through clinical history taking, interviews, and clinical examinations to exclude the presence of headache disorders, family history of migraine, or the presence of other neurological diseases.

#### 2.2.2. DNA isolation and genotyping

Buccal epithelial cells from the study subjects were collected using sterile swabs. Genomic DNA was isolated from these samples using a commercial nucleic acid extraction kit (Nucleospin Tissue; Macherey-Nagel GmbH & Co., KG, Düren, Germany), following the manufacturer's instructions. The DNA concentration and purity were assessed using a NanoDrop 2000 spectrophotometer (Thermo Scientific, USA). The extracted DNA was stored at −20 °C until further analysis. Genotyping of the investigated TNFa variant was performed using real-time polymerase chain reaction (PCR) on the LightCycler® 480 System (Roche Diagnostics, Germany) with SimpleProbe® probes (LightSNiP assays; TIB Molbiol, Berlin, Germany), followed by a melting curve analysis, as previously described [20]. More information regarding the genotyping methodology are accessible in the Supplementary File.

#### 2.2.3. Additional data extraction

Demographic information (age at the time of the investigation and sex) were collected for both groups. Moreover, for the migraine group, additional data were gathered: age of onset and migraine subtype (migraine with aura, migraine without aura, chronic migraine). Migraine subtypes were classified using the ICHD-3 criteria.

#### 2.2.4. Outcome measures and statistical analysis

The main objective of the study was to investigate the association between the TNFa gene polymorphism rs1800629 and overall migraine susceptibility. Secondary objectives were the association of rs1800629 TNFa gene variant with specific migraine subtypes—namely migraine with aura, migraine without aura, and chronic migraine—and assessing its potential effect on the age at migraine onset.

The Hardy-Weinberg equilibrium (HWE) was assessed among the HC using a chi-squared test implemented in the SPSS software (for version, see below). The power of our sample was estimated using CaTS Power Calculator for Genetic Studies (Center for Statistical Genetics, University of Michigan, Ann Arbor, Michigan, USA) [21]. The association between rs1800629 and migraines was analyzed using the SNPStats software, both in the overall sample and stratified by sex [22]. Multiple genetic models were evaluated, including dominant, recessive, co-dominant, over-dominant, and log-additive models.

Associations between TNFa rs1800629 and migraine subtypes were explored within the migraine cohort. Three separate analyses were performed, each considering a different outcome: migraine with aura versus the rest; migraine without aura versus the rest; and chronic migraine versus the rest. Once again, the SNPStats software was used. The potential impact of rs1800629 on the age at migraine onset was assessed using Cox proportional hazards regression models. Both unadjusted and sex-adjusted models were applied to account for possible confounding. Survival analyses were conducted using the IBM SPSS Statistics Software, version 26.0 (SPSS Inc., Chicago, IL, USA) for Windows.

A significance level of 5% (α = 0.05) was set throughout. The effect sizes were reported as Odds Ratios (OR, used to describe associations across genetic inheritance models) and Hazard Ratios (HR, used in Cox proportional hazards regression models), each accompanied by 95% Confidence Interval (95% CI).

### 2.3. Meta-analysis

Study reporting is in accordance with the Preferred Reporting Items for Systematic Reviews and Meta-Analyses (PRISMA) guidelines (Supplementary Table 1) [23]. This study was not pre-registered in an online database. All procedures were independently performed by two authors (I.L. and S.D.). Discrepancies were resolved by a third author (V.S.).

#### 2.3.1. Literature search

A systematic literature search was conducted using the MEDLINE database (via PubMed) and EMBASE (via Elsevier) to identify all relevant studies published up to April 2025 (date of final search update). The search strategy combined the following search terms across all searchable fields: (TNF-α OR “TNF α” OR TNFa OR “TNF alpha” OR TNF-a OR “TNF a” OR TNFa OR “Tumor necrosis factor-alpha” OR “Tumor necrosis factor alpha”) AND (rs1800629 OR 308G/A OR 308G>A OR 308 OR polymorphism OR variant OR gene) AND (migraine). In addition, the reference lists of all eligible articles, including relevant systematic reviews and meta-analyses, were manually screened to identify potentially relevant studies not captured through database searches. No additional search engines (e.g., Google Scholar or Semantic Scholar) were used.

#### 2.3.2. Eligibility criteria

Studies were included if they met the following criteria:

- Publication date up to April 2025;
- Designed as cross-sectional or case–control or cohort studies;
- Investigated TNFa rs1800629;
- Reported data on sample sizes, allelic, and/or genotypic frequencies for rs1800629; and
- Reported allele frequencies in HWE in the HC group

The exclusion criteria were as follows:

- Studies that assessed other SNPs;
- Studies that assessed other disorders;
- Non-observational studies (e.g., reviews, case-reports, letters to the editor, etc.);
- Studies that involved individuals younger than 18 years;
- Animal studies; and
- Non-English studies

Titles and abstracts were initially assessed for relevance. The full texts of the studies that qualified from the initial evaluation were reviewed for potential inclusion. If two or more publications featured overlapping participant groups, then the publication reporting the largest sample was included.

#### 2.3.3. Data extraction

Data were extracted according to standardized extraction forms: first author and publication year, country where the study took place, ethnicity of the participants, diagnostic criteria for migraine, laboratory technique for genotyping, numbers of patients and HC, demographics, allelic and/or genotypic frequencies for both groups. Data extraction was exclusively based on the information reported in the original publications. Studies with missing or incomplete data required for effect size estimation were excluded during the eligibility assessment, and no additional data retrieval strategies (e.g., contacting authors) were performed.

#### 2.3.4. Statistical analysis and quality assessment

The overall risk of migraine susceptibility was initially estimated for the minor allele (A versus G), which constituted the primary analysis. Subgroup analyses stratified by sex (men and women), race (Caucasians, Asians and African Americans), and migraine subtype (migraine with aura, migraine without aura) were also prespecified. Sequentially, the same analyses (primary and subgroup) were performed for the dominant (AA + AG versus GG), recessive (AA versus AG + GG), and over-dominant (AG versus AA + GG) models of inheritance. Data capitalization was based on the study reporting.

All statistical analyses were performed via RevMan, 8.13.0. For the allelic models, the significance threshold was conventionally set to α = 0.05 (P < 0.05). For the remaining analyses (models of inheritance), the stricter threshold of P ≤ 0.001 was adopted to account for multiple comparisons. Pooled estimates [ORs and 95% CIs] were estimated using the inverse variance of individual effects as weights. Forest plots were constructed to graphically depict the individual study and pooled estimates. The Q and I squared (*I*^2^) statistics were estimated to assess the statistical heterogeneity [24]. If PQ < 0.1 or *I*^2^ > 50%, then homogeneity was rejected [25]. If homogeneity was rejected, then the random-effects (RE) model was applied; otherwise, the fixed-effects (FE) model was used [26].

The quality of the retrieved case-control studies was assessed according to the Newcastle-Ottawa Scale (NOS). NOS evaluates nine methodological items and their reporting: 4 with respect to the participant selection, 2 for the comparability of groups, and 3 for the ascertainment of exposure/outcome. As a rule of thumb, values ≥7 are compatible with a good study quality, values from 4 to 6 are compatible with a moderate study quality, and values ≤3 are compatible with a poor study quality.

## 3. Results

### 3.1. Case-control study

A total of 123 individuals diagnosed with migraines (mean age: 44.4 ± 10.3 years; 18 males and 105 females) and an equal number of HC (mean age: 58.7 ± 12.0 years; 47 males and 76 females) were included in the study. The number of individuals excluded at each stage of recruitment was not systematically recorded. However, all participants were consecutively enrolled, and the predefined inclusion and exclusion criteria were consistently applied throughout the study. The genotype distribution of the TNFa rs1800629 polymorphism among the HC conformed to HWE (p = 1.00), thus indicating no deviation from the expected genetic variation. Assuming a minor allele frequency (MAF) of 16% in European populations [27] and an estimated prevalence of migraine equal to 15% [28], the study sample had approximately 80% statistical power to detect a significant association (α = 0.05) between the rs1800629 TNFa gene variant and migraines, thereby assuming a moderate relative risk of 1.70. Detailed demographic characteristics of the sample, along with allelic and genotypic frequencies, are presented in Table 1.

#### 3.1.1. Associations between TNFa rs1800629 and migraine

**Figure 1.**
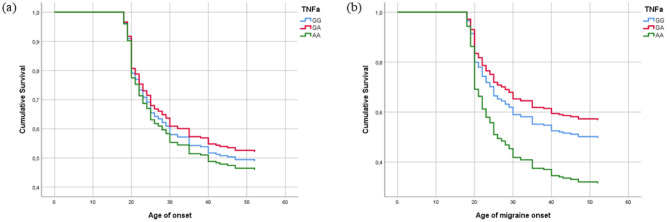
The association of TNFa (tumour necrosis factor a) with age of migraine onset. (a) unadjusted and (b) sex-adjusted Cox proportional hazards models. Note: (a) GA vs. GG: Hazard ratio = 0.91 (0.54–1.54); AA vs. GG Hazard ratio = 1.09 (0.15–7.79); (b) GA vs. GG: Hazard ratio = 0.81 (0.48–1.37); AA vs. GG Hazard ratio = 1.65 (0.23–11.96).

No statistically significant associations were observed between the rs1800629 TNFa gene polymorphism and migraine susceptibility under any examined genetic mode of inheritance. Specifically, the OR and corresponding 95% CI were as follows: log-additive model OR = 0.85 (95% CI: 0.44–1.62), P = 0.62; over-dominant model OR = 0.82 (95% CI: 0.40–1.68), P = 0.58; recessive model OR = 1.00 (95% CI: 0.06–16.17), P = 1.00; dominant model OR = 0.83 (95% CI: 0.41–1.67), P = 0.59; and co-dominant models OR₁ = 0.82 (95% CI: 0.40–1.68) and OR₂ = 0.97 (95% CI: 0.06–15.74), omnibus P = 0.86 (Table 2). Likewise, subgroup analyses stratified by sex revealed no significant associations in either the male or female participants (Table 2). Additionally, a survival analysis using Cox proportional hazards models—both unadjusted and adjusted for sex—demonstrated no significant effect of rs1800629 on the age at migraine onset (Figure 1). Regarding migraine subtypes, no significant associations were detected between rs1800629 and migraine with aura, migraine without aura, or chronic migraine (Table 3).

### 3.2. Meta-analysis

**Figure 2.**
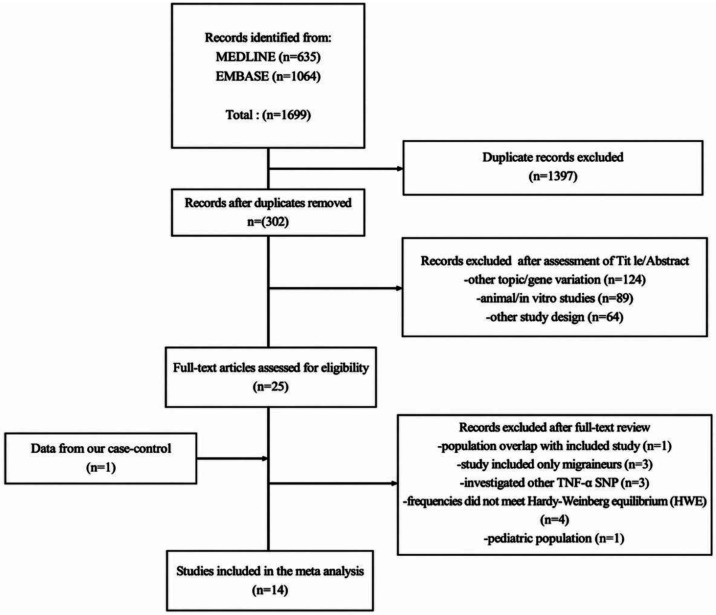
Study selection flowchart.

The literature search yielded a total of 1699 studies, with 635 from MEDLINE (via PubMed) and 1064 from EMBASE (via Elsevier). After the title and abstract screening, 25 full-text articles were assessed for eligibility. Of these, 19 studies were deemed eligible for inclusion in this systematic review, including the present study [17],[29]–[45]. The study quality assessment is summarized in Supplementary Table 2, with no studies rated as poor quality. Upon further scrutiny, 4 studies were excluded due to deviation from HWE (P < 0.05), and another study was excluded because it involved participants with an age lower than 18 years. Consequently 14 studies, including the current case-control study, were included in the quantitative analysis, as depicted in Figure 2. The study characteristics can be found in Table 4.

**Figure 3.**
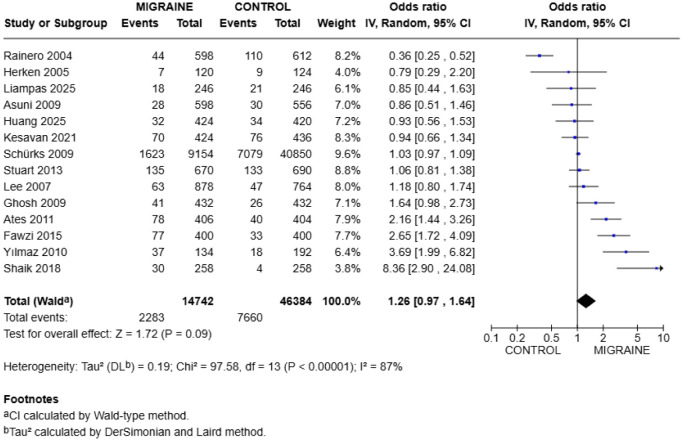
Association between TNFa rs1800629 and migraines – allelic model.

**Figure 4.**
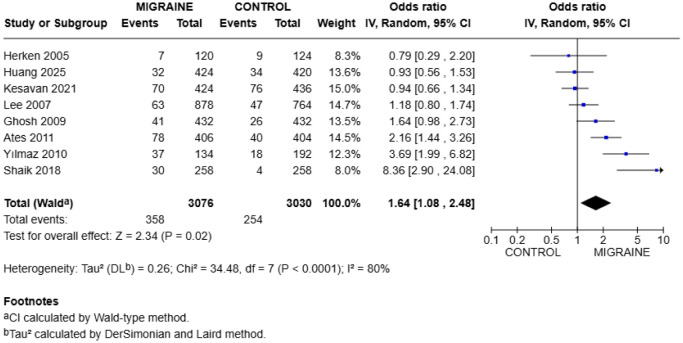
Association between TNFa rs1800629 and migraines in individuals of Asian ancestry – allelic model.

**Figure 5.**
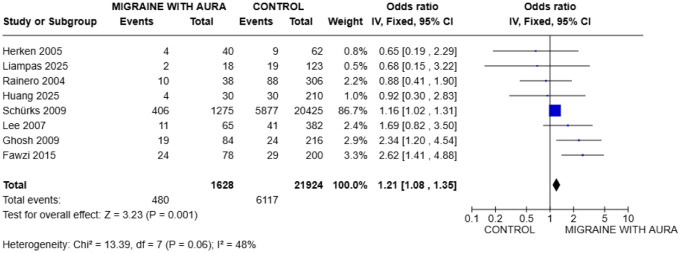
Association between rs1800629 and migraines with aura – over-dominant model.

The main meta-analysis indicated a non-significant trend toward association for the rs1800629 TNFa polymorphism [OR = 1.26, 95% CI = (0.97–1.64), P = 0.09] (Figure 3). However, substantial heterogeneity was observed (I² = 87%), thus suggesting considerable variability across the analyzed studies and warranting a cautious interpretation of these findings. Subgroup analyses by race captured a significant association for individuals of Asian but not Caucasian ancestry, which might, at least in part, explain the aforementioned heterogeneity [OR = 1.64, 95% CI = (1.08–2.48), P = 0.02] (Figure 4). Subgroup analyses based on the migraine phenotype favored stronger associations between the rs1800629 TNFa gene variant and migraine with aura. Specifically, a statistically significant association was only observed for the over-dominant model of inheritance in migraines with aura [OR = 1.21, 95% CI = (1.08–1.35), P = 0.001] (Figure 5). On the other hand, substantial heterogeneity was observed on the dominant model of inheritance, which could likely be attributed to imprecise, albeit stronger, effect estimates. This pattern may be influenced by the extremely low prevalence of homozygosity for the minor allele, thus suggesting a potential heterozygote-driven effect; however, these findings should be interpreted with caution. Subgroup analyses by sex were insignificant. The secondary meta-analyses (for the dominant, recessive, and over-dominant models) were also insignificant (Table 5). The subgroup and secondary meta-analyses are provided in detail in the Supplementary material.

**Table 1.** Participants characteristics, allelic and genotypic frequencies of TNFa rs1800629.

Parameter	Migraine (N = 123)		Healthy controls (N = 123)		p value	
Age (mean years ± SD)	44.4 ± 10.3		58.7 ± 12.0		P < 0.001	
Sex (Male/%)	18 (14.6%)		47 (38.2%)		P < 0.001	
Age of migraine onset (mean years ± SD)	25.1 ± 7.5		NA		NA	
Migraine subtype (with aura/ without aura/chronic)	18/68/37 (14.6/55.3/30.1%)		NA		NA	
Genotypes	Migraine (N = 123)		Healthy controls (N = 123)		Total participants (N = 246)	
A/A	1	0.01	1	0.01	2	0.01
G/A	16	0.13	19	0.15	35	0.14
G/G	106	0.86	103	0.84	209	0.85
Alleles			
A	18	0.07	21	0.09	39	0.08
G	228	0.93	225	0.91	453	0.92

Note: N: number of participants; NA: non-applicable; SD: standard deviation; TNFa: tumour necrosis factor alpha

**Table 2.** Single locus association of TNFa rs1800629 with migraine.

	Mode of Inheritance	Genotype	Odds Ratio (95% CI)	P value
Whole sample	Co-dominant	G/G	1.00	0.86
		G/A	0.82 (0.40–1.68)	
		A/A	0.97 (0.06–15.74)	
	Dominant	G/G	1.00	0.59
		G/A–A/A	0.83 (0.41–1.67)	
	Recessive	G/A–G/G	1.00	1.00
		A/A	1.00 (0.06–16.17)	
	Over-dominant	G/G–A/A	1.00	0.58
		G/A	0.82 (0.40–1.68)	
	Log-additive	-	0.85 (0.44–1.62)	0.62

Women	Co-dominant	G/G	1.00	0.37
		G/A	0.69 (0.31–1.50)	
		A/A	NA (0.00–NA)	
	Dominant	G/G	1.00	0.43
		G/A–A/A	0.73 (0.34–1.59)	
	Recessive	G/A–G/G	1.00	0.30
		A/A	NA (0.00–NA)	
	Over-dominant	G/G–A/A	1.00	0.33
		G/A	0.68 (0.31–1.49)	
	Log-additive	-	0.80 (0.38–1.67)	0.33

Men	Co-dominant	G/G	1.00	0.66
		G/A	0.62 (0.06–5.93)	
		A/A	0.00 (0.00–NA)	
	Dominant	G/G	1.00	0.51
		G/A–A/A	0.49 (0.05–4.55)	
	Recessive	G/A–G/G	1.00	0.42
		A/A	0.00 (0.00–NA)	
	Over-dominant	G/G–A/A	1.00	0.68
		G/A	0.63 (0.07–6.07)	
	Log-additive	-	0.48 (0.07–3.61)	0.43

Note: TNFa: tumour necrosis factor alpha; CI; confidence interval; NA: applicable (due to the one observation with AA genotype, per group).

**Table 3.** Single locus association of TNFa rs1800629 with migraine subtypes.

	Mode of Inheritance	Genotype	Odds Ratio (95% CI)	P value
Chronic migraine	Co-dominant	G/G	1.00	0.70
		G/A	1.05 (0.34–3.27)	
		A/A	0.00 (0.00–NA)	
	Dominant	G/G	1.00	0.95
		G/A–A/A	0.96 (0.31–2.96)	
	Recessive	G/A–G/G	1.00	0.40
		A/A	0.00 (0.00–NA)	
	Over-dominant	G/G–A/A	1.00	0.91
		G/A	1.07 (0.34–3.32)	
	Log-additive	-	0.89 (0.31–2.54)	0.83

Migraine with aura	Co-dominant	G/G	1.00	0.82
		G/A	1.24 (0.26–6.01)	
		A/A	NA (0.00–NA)	
	Dominant	G/G	1.00	0.71
		G/A–A/A	1.33 (0.28–6.40)	
	Recessive	G/A–G/G	1.00	0.57
		A/A	NA (0.00–NA)	
	Over-dominant	G/G–A/A	1.00	0.79
		G/A	1.23 (0.26–5.94)	
	Log-additive	-	1.39 (0.31–6.16)	0.66

Migraine without aura	Co-dominant	G/G	1.00	0.55
		G/A	1.06 (0.37–3.07)	
		A/A	NA (0.00–NA)	
	Dominant	G/G	1.00	0.75
		G/A–A/A	1.18 (0.42–3.34)	
	Recessive	G/A–G/G	1.00	0.27
		A/A	NA (0.00–NA)	
	Over-dominant	G/G–A/A	1.00	0.93
		G/A	1.05 (0.36–3.02)	
	Log-additive	-	1.28 (0.49–3.37)	0.61

Note: TNFa: tumour necrosis factor alpha; CI; confidence interval; NA: applicable (due to the one observation with AA genotype, per group).

**Table 4.** Study characteristics and genotypic frequencies of TNFa rs1800629.

Author - Publication year	Diagnosis	Cases	Controls	Source of controls	Country	Race	Genotyping Method	Cases			Controls			HWE
								GG	GA	AA	GG	GA	AA	
Trabace 2002 [29]	HIS	79	101	PB	Italy	Caucasian	PCR–RFLP	67	12	0	90	9	2	P < 0.05
Rainero 2004 [30]	HIS	299	306	HB	Italy	Caucasian	PCR–RFLP	256	42	1	207	88	11	0.858
Herken 2005 [45]	HIS	60	62	HB	Turkey	Asian	PCR–RFLP	54	5	1	53	9	0	0.858
Mazaheri 2006[31]	HIS	221	183	HB	Iran	Asian	PCR-SSP	51	163	7	94	86	3	P < 0.05
Lee 2007 [33]	HIS	439	382	HB	Korea	Asian	PCR	377	61	1	338	41	3	0.3849
Ghosh 2009 [32]	HIS	216	216	HB	India	Asian	PCR–RFLP	175	41	0	191	24	1	0.858
Asuni 2009 [35]	ICHD-II	299	278	HB	Italy	Caucasian	PCR	272	26	1	249	28	1	0.858
Schürks 2009[38]	SR	4577	20425	PB	USA	Caucasian	MASSarray	3081	1369	127	13947	5877	601	0.858
Pappa 2010 [36]	ICHD-II	103	178	HB	Greece	Caucasian	PCR–RFLP	89	14	0	145	31	2	0.858
Yilmaz 2010 [37]	ICHD-II	67	96	HB	Turkey	Asian	PCR–RFLP	37	23	7	79	16	1	0.858
Ates 2011 [34]	HIS	203	202	HB	Turkey	Asian	ARMS-PCR	125	78	0	162	40	0	0.3155
Stuart 2013 [40]	HIS	335	345	HB	Australia	Caucasian	HRM-RFLP	220	95	20	230	97	18	0.292
Fawzi 2015 [39]	HIS	200	200	HB	Egypt	Egyptian	PCR–RFLP	136	51	13	169	29	2	0.858
Shaik 2018 [41]	N/A	129	129	HB	Malaysia	Asian	PCR–RFLP	99	30	0	125	4	0	0.858
Hamad 2021 [42]	HIS	183	184	PB	Jordan	Asian	PCR–RFLP	96	72	15	51	108	25	P < 0.05
Kesavan 2021 [17]	HIS	212	218	HB	India	Asian	ARMS-PCR	158	38	16	152	56	10	0.3155
Tatlısuluoğlu 2021 [43]	HIS	70	65	HB	Turkey	Asian	PCR–RFLP	2	38	30	0	52	13	P < 0.05
Huang 2025 [44]	ICHD-II	212	210	HB	China	Asian	PCR-SNaPshot	180	32	0	178	30	2	P > 0.05
Liampas 2026 [current study]	ICHD-III	123	123	HB	Greece	Caucasian	PCR	106	16	1	103	19	1	1

Note: TNFa: tumour necrosis factor alpha; HIS: International Headache Society; ICHD: International Classification of Headache Disorder; SR: Self-Reported; N/A: Not Available; PB: Population Based; HB: Hospital Based; PCR: Polymerase Chain Reaction; PCR–RFLP: Polymerase Chain Reaction-Restriction Fragment Length Polymorphism; ARMS-PCR: Amplification-Refractory Mutation System- Polymerase Chain Reaction; HWE: Hardy-Weinberg equilibrium.

**Table 5.** Association between TNFa rs1800629 and migraine, main and subgroup analyses.

Group	Genetic Model	Comparison	OR	95% CI	p-value	I² (%)	Model	Number of Studies
Overall	Allelic	A vs. G	1.26	0.97–1.64	0.09	87	RE	14
	Recessive	AA vs. GG + GA	1.14	0.58–2.25	0.70	68	RE	
	Dominant	GA + AA vs. GG	1.27	0.96–1.70	0.10	86	RE	
	Over-dominant	GA vs. GG + AA	1.22	0.93–1.59	0.16	83	RE	
MA	Allelic	A vs. G	1.28	0.86–1.90	0.22	70	RE	8
	Recessive	AA vs. GG + GA	1.19	0.88–1.61	0.26	9	FE	
	Dominant	GA + AA vs. GG	1.35	0.91–2.00	0.13	64	RE	
	Over-dominant	GA vs. GG + AA	1.21	1.08–1.35	0.001	48	FE	
MO	Allelic	A vs. G	1.11	0.78–1.58	0.57	85	RE	10
	Recessive	AA vs. GG + GA	1.22	0.46–3.23	0.70	61	RE	
	Dominant	GA + AA vs. GG	1.08	0.75–1.56	0.66	83	RE	
	Over-dominant	GA vs. GG + AA	1.04	0.75–1.42	0.83	75	RE	
Female	Allelic	A vs. G	1.08	0.71–1.63	0.72	86	RE	7
	Recessive	AA vs. GG + GA	0.94	0.78–1.14	0.53	37	FE	
	Dominant	GA + AA vs. GG	1.08	0.71–1.65	0.72	84	RE	
	Over-dominant	GA vs. GG + AA	1.06	0.72–1.55	0.78	79	RE	
Male	Allelic	A vs. G	0.71	0.45–1.13	0.15	49	FE	5
	Recessive	AA vs. GG + GA	0.70	0.17–2.94	0.63	0	FE	
	Dominant	GA + AA vs. GG	0.71	0.42–1.18	0.18	44	FE	
	Over-dominant	GA vs. GG + AA	0.76	0.45–1.29	0.31	10	FE	
Asian	Allelic	A vs. G	1.64	1.08–2.48	0.02	80	RE	8
	Recessive	AA vs. GG + GA	1.54	0.85–2.80	0.15	40	FE	
	Dominant	GA + AA vs. GG	1.70	1.06–2.72	0.03	81	RE	
	Over-dominant	GA vs. GG + AA	1.60	0.99–2.60	0.06	81	RE	
Caucasian	Allelic	A vs. G	0.79	0.55–1.14	0.20	87	RE	5
	Recessive	AA vs. GG + GA	0.94	0.78–1.13	0.50	26	FE	
	Dominant	GA + AA vs. GG	0.78	0.53–1.16	0.22	86	RE	
	Over-dominant	GA vs. GG + AA	0.80	0.56–1.15	0.23	81	RE	

Note: TNFa: tumour necrosis factor alpha; MA: Migraine with aura, MO: Migraine without aura, OR: Odds Ratio, CI: Confidence Interval, RE: Random effects model, FE: Fixed effects model.

## 4. Discussion

In the current study, we examined the potential association between TNFa 308G>A and migraines. This was achieved through a case-control study that involved individuals with Greek ancestry, complemented by a meta-analysis of published evidence that incorporated our findings. Our results were consistent with previous meta-analyses that reported a significant association between rs1800629 and migraines in Asian populations [18],[46],[47]. Subgroup analyses based on sex and migraine subtype favored stronger associations between rs1800629 and migraines with aura, according to the over-dominant model of inheritance.

The rs1800629 polymorphism has been associated with increased TNFa expression. TNFa is a pro-inflammatory cytokine implicated in pain-signaling and neuroinflammation [48]–[50]. It has been linked to the sensitization of trigeminal nociceptive neurons and the upregulation of calcitonin gene-related peptide (CGRP), both of which are considered key processes in the pathophysiology of migraines [51],[52]. Additionally, TNFa may contribute to glutamate excitotoxicity, thereby stimulating glutamate release and N-Methyl-D-Aspartate Receptor (NMDAR) activation, and resulting in calcium influx and neuronal injury [50],[53]. In this context, TNFa could play a role in the theoretical neurophysiological correlation of aura (i.e., cortical spreading depression [54],[55]). In our analysis, subgroup testing captured an association between rs1800629 and the subgroup of migraines with aura. Nevertheless, this observation should be interpreted with caution and requires confirmation in larger, well-powered studies.

Racial heterogeneity in the association of rs1800629 with migraine susceptibility implies that the association pattern is more complex than a direct risk-conferring effect of TNFa. For example, non-random associations with nearly loci that are themselves related to migraines might underlie associations in Asian populations (linkage disequilibrium). Moreover, distinct racial associations may reflect underlying ancestral differences rather than true disease susceptibility (population stratification theory) [56]. Of note, racial differences may be an outcome of epigenetic modifications [57]. Environmental factors that potentially vary across populations may interact with gene expression and lead to important phenotypic disparities.

### 4.1. Limitations, strengths, and future perspectives

It is important to acknowledge the limitations of our study. First, the statistical power of our case-control study was just slightly above 80%, meaning there is a 20% probability that we could have missed true associations. Considering that the effect size yielded by the meta-analysis was small, the likelihood of missing a true association is even greater. Second, our analysis did not account for a variety of genetic and environmental factors, which means that unmeasured or unknown confounders could have influenced the results. Additionally, the participants in our study were habitants of a restricted geographical region in Greece. While this could have unintentionally led to some degree of consistency in the environmental exposures, it also likely introduced a genetic bias that arised from the homogeneity of the population. This overmatching could have impacted the generalizability of our findings, especially regarding genetic variations. The performance of a meta-analysis resolves both of the aforementioned issues. However, not all sources of statistical heterogeneity could be fully identified, and the studies were quite heterogeneous in undermining the precision of our estimates. Variability in racial backgrounds and differences in control selection (hospital-based instead of community-based controls were often enrolled) have probably introduced an important amount of heterogeneity. Apart from the aforementioned parameters, heterogeneity may have been inflated by phenotypic differences among the migraine groups (the proportion of individuals with aura per study, the proportion of men and women per study, age groups, the frequency and severity of symptoms, and so on). Unfortunately, incomplete reporting in several studies limited our ability to fully capitalize on the published data in the context of detailed subgroup analyses [58],[59].

This study has several strengths as well, including the combined use of a case–control design and an updated meta-analysis, the inclusion of a well-characterized and ethnically homogeneous population, and the application of standardized diagnostic criteria (ICHD-3). The evaluation of multiple genetic models and clinically relevant subgroup analyses enhances the robustness of the findings of our study. Moreover, the incorporation of recent studies and the assessment of heterogeneity strengthen the validity of the meta-analysis.

Future research should focus on large-scale, multicenter studies with well-characterized populations to further clarify the role of the rs1800629 TNFa genetic variant in migraine susceptibility, particularly across different ethnic groups and migraine subtypes and sub-phenotypes. Given the potential indication of a heterozygote-driven effect, additional studies that explore genotype-specific functional consequences and gene–environment interactions are warranted. Integrating genomic data with transcriptomic and proteomic approaches may help elucidate the biological mechanisms that underlie the observed associations. Finally, investigating the interaction between TNFa genetic variants and emerging therapeutic targets (e.g. CGRP-related pathways), could provide valuable insights into personalized approaches for the management of patients with migraine.

## 5. Conclusions

We reported a significant association between rs1800629 and migraines in Asian but not Caucasian populations. These findings support a population-specific genetic effect and reinforce the importance of considering ethnic heterogeneity in the migraine genetic pathophysiological background. Further research is needed to confirm the absence of this association in non-Asian populations, as well as to shed more light in the potentially more remarkable affinity of rs1800629 with migraines with aura.

## Use of AI tools declaration

The authors declare they have not used Artificial Intelligence (AI) tools in the creation of this article.



## References

[1] Liampas IN, Siokas V, Aloizou AM (2020). Pyridoxine, folate and cobalamin for migraine: A systematic review. Acta Neurol Scand.

[2] Siokas V, Liampas I, Aloizou AM (2022). Deciphering the role of the rs2651899, rs10166942, and rs11172113 polymorphisms in migraine: a meta-analysis. Medicina (Kaunas).

[3] Yeh PK, An YC, Hung KS (2024). Influences of genetic and environmental factors on chronic migraine: a narrative review. Curr Pain Headache Rep.

[4] Dong L, Dong W, Jin Y (2025). The global burden of migraine: a 30-year trend review and future projections by age, sex, country, and region. Pain Ther.

[5] Husøy AK, Yu S, Liu R (2025). The global prevalence of headache disorders of public-health importance: a meta-analysis of population-based individual participant data from 41,614 adults from 17 countries. J Headache Pain.

[6] Hautakangas H, Winsvold BS, Ruotsalainen SE (2022). Genome-wide analysis of 102,084 migraine cases identifies 123 risk loci and subtype-specific risk alleles. Nat Genet.

[7] Torok D, Petschner P, Baksa D (2024). Improved polygenic risk prediction in migraine-first patients. J Headache Pain.

[8] Frimpong-Manson K, Ortiz YT, McMahon LR (2024). Advances in understanding migraine pathophysiology: a bench to bedside review of research insights and therapeutics. Front Mol Neurosci.

[9] Liampas I, Siokas V, Bakirtzis C, Martin CR, Patel VB, Preedy VR (2023). Chapter 19 - Vitamin B12, folate, and migraine. Vitamins and Minerals in Neurological Disorders.

[10] Sudershan A, Sudershan S, Sharma I (2024). Role of TNF-α in the pathogenesis of migraine. Pain Res Manag.

[11] TNF tumor necrosis factor [Homo sapiens (human)]. (cited 2025 March 07).

[12] Chen L, Huang Z, Liao Y (2019). Association between tumor necrosis factor polymorphisms and rheumatoid arthritis as well as systemic lupus erythematosus: a meta-analysis. Braz J Med Biol Res.

[13] Popa C, Netea MG, van Riel PL (2007). The role of TNF-alpha in chronic inflammatory conditions, intermediary metabolism, and cardiovascular risk. J Lipid Res.

[14] Lio D, Annoni G, Licastro F (2006). Tumor necrosis factor-alpha-308A/G polymorphism is associated with age at onset of Alzheimer's disease. Mech Ageing Dev.

[15] Sudershan A, Sudershan S, Behlam I (2025). TNF-α-308 G > A polymorphism (rs1800629) and migraine susceptibility: a case–control study with updated meta-analysis. Nucleus.

[16] Fan W, Maoqing W, Wangyang C (2011). Relationship between the polymorphism of tumor necrosis factor-α-308 G>A and susceptibility to inflammatory bowel diseases and colorectal cancer: a meta-analysis. Eur J Hum Genet.

[17] Kesavan P, Satheesh AP, Husain R (2021). Genetic predisposition of TNFα gene polymorphism in South-Indian Migraineurs and meta-analysis. Front Biosci (Elite Ed).

[18] Sudershan A, Sudershan S, Younis M (2023). Enlightening the association between TNF-α-308 G > A and migraine: a meta-analysis with meta-regression and trial sequential analysis. BMC Neurol.

[19] Tsirelis D, Tsekouras A, Stamati P (2024). The impact of genetic factors on the response to migraine therapy. Rev Neurosci.

[20] Papasavva M, Vikelis M, Siokas V (2022). Variability in oxidative stress-related genes (SOD2, CAT, GPX1, GSTP1, NOS3, NFE2L2, and UCP2) and susceptibility to migraine clinical phenotypes and features. Front Neurol.

[21] Skol AD, Scott LJ, Abecasis GR (2006). Joint analysis is more efficient than replication-based analysis for two-stage genome-wide association studies. Nat Genet.

[22] Solé X, Guinó E, Valls J (2006). SNPStats: a web tool for the analysis of association studies. Bioinformatics.

[23] Page MJ, McKenzie JE, Bossuyt PM (2021). The PRISMA 2020 statement: an updated guideline for reporting systematic reviews. BMJ.

[24] Liampas I, Siokas V, Brotis A (2020). Endogenous melatonin levels and therapeutic use of exogenous melatonin in migraine: systematic review and meta-analysis. Headache.

[25] Liampas I, Siokas V, Brotis A (2020). Vitamin D serum levels in patients with migraine: A meta-analysis. Rev Neurol (Paris).

[26] Liampas I, Siokas V, Mentis AA (2020). Serum homocysteine, pyridoxine, folate, and vitamin b12 levels in migraine: systematic review and meta-analysis. Headache.

[27] rs1800629 Ref SNP Report-dbSNP.

[28] Coppola G, Brown JD, Mercadante AR (2025). The epidemiology and unmet need of migraine in five european countries: results from the national health and wellness survey. BMC Public Health.

[29] Trabace S, Brioli G, Lulli P (2002). Tumor necrosis factor gene polymorphism in migraine. Headache.

[30] Rainero I, Grimaldi LM, Salani G (2004). Association between the tumor necrosis factor-alpha -308 G/A gene polymorphism and migraine. Neurology.

[31] Mazaheri S, Hajilooi M, Rafiei A (2006). The G-308A promoter variant of the tumor necrosis factor-alpha gene is associated with migraine without aura. J Neurol.

[32] Ghosh J, Joshi G, Pradhan S (2010). Investigation of TNFA 308G > A and TNFB 252G > A polymorphisms in genetic susceptibility to migraine. J Neurol.

[33] Lee KA, Jang SY, Sohn KM (2007). Association between a polymorphism in the lymphotoxin-a promoter region and migraine. Headache.

[34] Ates O, Kurt S, Altinisik J (2011). Genetic variations in tumor necrosis factor alpha, interleukin-10 genes, and migraine susceptibility. Pain Med.

[35] Asuni C, Stochino ME, Cherchi A (2009). Migraine and tumour necrosis factor gene polymorphism. An association study in a Sardinian sample. J Neurol.

[36] Pappa S, Hatzistilianou M, Kouvatsi A (2010). Tumour necrosis factor gene polymorphisms and migraine in Greek children. Arch Med Sci.

[37] Yilmaz IA, Ozge A, Erdal ME (2010). Cytokine polymorphism in patients with migraine: some suggestive clues of migraine and inflammation. Pain Med.

[38] Schürks M, Kurth T, Buring JE (2009). A candidate gene association study of 77 polymorphisms in migraine. J Pain.

[39] Fawzi MS, El-Shal AS, Rashad NM (2015). Influence of tumor necrosis factor alpha gene promoter polymorphisms and its serum level on migraine susceptibility in Egyptian patients. J Neurol Sci.

[40] Stuart S, Maher BH, Sutherland H (2013). Genetic variation in cytokine-related genes and migraine susceptibility. Twin Res Hum Genet.

[41] Shaik MM, Abubakar MB, Tan HL (2018). Influence of TNF-α and ESR1 polymorphisms on vascular, hormonal and inflammatory biomarkers in migraine. J Med Sci.

[42] Hamad N, Alzoubi KH, Swedan SF (2021). Association between tumor necrosis factor alpha and lymphotoxin alpha gene polymorphisms and migraine occurrence among Jordanians. Neurol Sci.

[43] Tatlısuluoğlu D, Derle E, Ocal R (2021). The relationship between tumor necrosis factor α-308 G/A poly-morphism and serum tumor necrosis factor α levels in patients with migraine without aura. Troia Med J.

[44] Huang G, Dong X, Shao X (2025). Correlation of tumor necrosis factor-α and interleukin-1 single-nucleotide polymorphisms with the risk of migraine development. Front Genet.

[45] Herken H, Erdal ME, Yilmaz M (2005). The -308 G/A polymorphism of tumor necrosis factor alpha gene is not associated with migraine. Pain Clinic.

[46] Chen M, Tang W, Hou L (2015). Tumor necrosis factor (TNF) -308G>A, nitric oxide synthase 3 (NOS3) +894G>T polymorphisms and migraine risk: a meta-analysis. PLoS One.

[47] Schürks M, Rist PM, Zee RY (2011). Tumour necrosis factor gene polymorphisms and migraine: a systematic review and meta-analysis. Cephalalgia.

[48] Duan YW, Chen SX, Li QY (2022). Neuroimmune mechanisms underlying neuropathic pain: the potential role of TNF-α-necroptosis pathway. Int J Mol Sci.

[49] Miller F, Fenart L, Landry V (2005). The MAP kinase pathway mediates transcytosis induced by TNF-alpha in an in vitro blood-brain barrier model. Eur J Neurosci.

[50] Gonzalez Caldito N (2023). Role of tumor necrosis factor-alpha in the central nervous system: a focus on autoimmune disorders. Front Immunol.

[51] Durham ZL, Hawkins JL, Durham PL (2017). Tumor necrosis factor-Alpha stimulates cytokine expression and transient sensitization of trigeminal nociceptive neurons. Arch Oral Biol.

[52] Bowen EJ, Schmidt TW, Firm CS (2006). Tumor necrosis factor-alpha stimulation of calcitonin gene-related peptide expression and secretion from rat trigeminal ganglion neurons. J Neurochem.

[53] Olmos G, Lladó J (2014). Tumor necrosis factor alpha: a link between neuroinflammation and excitotoxicity. Mediators Inflamm.

[54] Biscetti L, Cresta E, Cupini LM (2023). The putative role of neuroinflammation in the complex pathophysiology of migraine: From bench to bedside. Neurobiol Dis.

[55] Charles AC, Baca SM (2013). Cortical spreading depression and migraine. Nat Rev Neurol.

[56] Hellwege JN, Keaton JM, Giri A (2017). Population stratification in genetic association studies. Curr Protoc Hum Genet.

[57] Gallardo VJ, Vila-Pueyo M, Pozo-Rosich P (2023). The impact of epigenetic mechanisms in migraine: Current knowledge and future directions. Cephalalgia.

[58] Kodounis M, Liampas IN, Constantinidis TS (2020). Assessment of the reporting quality of double-blind RCTs for ischemic stroke based on the CONSORT statement. J Neurol Sci.

[59] Liampas I, Chlinos A, Siokas V (2019). Assessment of the reporting quality of RCTs for novel oral anticoagulants in venous thromboembolic disease based on the CONSORT statement. J Thromb Thrombolysis.

