# Supplementary material for: Association between TNFa rs1800629 and migraine: Case-control study and updated meta-analysis
Source: AIMS Neurosci. 2026 May 29;13(2):295–315. doi: 10.3934/Neuroscience.2026013 (PMC13366164; doi:10.3934/Neuroscience.2026013)
Supplement: Supplementary file 1 [file neurosci-13-02-013-s001.pdf]

Neuroscience

AIMS Neuroscience, 13(2): 295–315.
DOI: 10.3934/Neuroscience.2026013
Received: 12 December 2025
Revised: 11 April 2026
Accepted: 22 April 2026
Published: 29 May 2026

https://www.aimspress.com/journal/neuroscience

Research article

Association between TNFa rs1800629 and migraine: Case-control study and updated meta-analysis

Ioannis Liampas[1,2], Maria Papasavva[3,4], Silvia Demiri[5], Michail Vikelis[6], Chrysoula Marogianni[1,2], Evripidis Vlachakis[1,2], Emmanouil V Dermitzakis[7], Aristidis Tsatsakis[8,9,10], Nikolaos Drakoulis[4], Efthimios Dardiotis[1,2,*,†] and Vasileios Siokas[1,2,*,†]

[1] Department of Neurology, University Hospital of Larissa, Faculty of Medicine, University of Thessaly, Mezourlo Hill, 41110 Larissa, Greece
[2] Laboratory of Neurogenetics, University of Thessaly, Mezourlo Hill, 41110 Larissa, Greece
[3] Department of Pharmacy, School of Health Sciences, Frederick University, Nicosia, Cyprus
[4] Research Group of Clinical Pharmacology and Pharmacogenomics, Faculty of Pharmacy, School of Health Sciences, National and Kapodistrian University of Athens, Panepistimiopolis Zografou, 15771, Athens, Greece
[5] School of Medicine, University of Patras, Rio Patras 26504, Greece
[6] Headache Clinic, Mediterraneo Hospital, 166 75 Glifada, Greece
[7] Euromedica General Clinic, 546 45 Thessaloniki, Greece
[8] Center of Toxicology & Science Applications, Medical School, University of Crete, 71003 Heraklion, Greece
[9] Universidad Ecotec, Km. 13.5 Samborondon, EC 092302 Samborondon, Ecuador
[10] Sechenov IM, First State Medical University, 119991 Moscow, Russia

* **Correspondence:** Efthimios Dardiotis: Email: edar@med.uth.gr; Vasileios Siokas: Email: vsiokas@med.uth.gr; Tel: + 30 6972437386.

† Shared senior authorship.

Supplementary

Genotyping of the investigated TNFa SNP was conducted using real-time Polymerase Chain Reaction in a LightCycler® 480 System (Roche Diagnostics, Germany) with SimpleProbe® probes (LightSNiP assay; TIB Molbiol, Berlin, Germany), according to the manufacturer's instructions. Particularly, all reactions were per-formed in a total volume of 20 μL, using approximately 50 ng of DNA samples. The PCR protocol included an initial denaturation step at 95 °C for 10 minutes, followed by 45 cycles of 95 °C for 10 seconds, 60 °C for 10 seconds, and 72 °C for 15 seconds. Melting curve analysis followed, and the corresponding genotypes were determined according to characteristic melting curves.

Supplementary Table 1. Prisma 2020 checklist.

Section and Topic	Item #	Checklist item	Location where item is reported
**TITLE**			
Title	1	Identify the report as a systematic review.	1
**ABSTRACT**			
Abstract	2	See the PRISMA 2020 for Abstracts checklist.	2
**INTRODUCTION**			
Rationale	3	Describe the rationale for the review in the context of existing knowledge.	3
Objectives	4	Provide an explicit statement of the objective(s) or question(s) the review addresses.	3
**METHODS**			
Eligibility criteria	5	Specify the inclusion and exclusion criteria for the review and how studies were grouped for the syntheses.	6,7
Information sources	6	Specify all databases, registers, websites, organisations, reference lists and other sources searched or consulted to identify studies. Specify the date when each source was last searched or consulted.	6
Search strategy	7	Present the full search strategies for all databases, registers and websites, including any filters and limits used.	6
Selection process	8	Specify the methods used to decide whether a study met the inclusion criteria of the review, including how many reviewers screened each record and each report retrieved, whether they worked independently, and if applicable, details of automation tools used in the process.	6,7
Data collection process	9	Specify the methods used to collect data from reports, including how many reviewers collected data from each report, whether they worked independently, any processes for obtaining or confirming data from study investigators, and if applicable, details of automation tools used in the process.	6,7
Data items	10a	List and define all outcomes for which data were sought. Specify whether all results that were compatible with each outcome domain in each study were sought (e.g. for all measures, time points, analyses), and if not, the methods used to decide which results to collect.	7
	10b	List and define all other variables for which data were sought (e.g. participant and	7

		intervention characteristics, funding sources). Describe any assumptions made about any missing or unclear information.	
Study risk of bias assessment	11	Specify the methods used to assess risk of bias in the included studies, including details of the tool(s) used, how many reviewers assessed each study and whether they worked independently, and if applicable, details of automation tools used in the process.	7
Effect measures	12	Specify for each outcome the effect measure(s) (e.g. risk ratio, mean difference) used in the synthesis or presentation of results.	7
Synthesis methods	13a	Describe the processes used to decide which studies were eligible for each synthesis (e.g. tabulating the study intervention characteristics and comparing against the planned groups for each synthesis (item #5)).	6,7
	13b	Describe any methods required to prepare the data for presentation or synthesis, such as handling of missing summary statistics, or data conversions.	N/A
	13c	Describe any methods used to tabulate or visually display results of individual studies and syntheses.	7
	13d	Describe any methods used to synthesize results and provide a rationale for the choice(s). If meta-analysis was performed, describe the model(s), method(s) to identify the presence and extent of statistical heterogeneity, and software package(s) used.	7
	13e	Describe any methods used to explore possible causes of heterogeneity among study results (e.g. subgroup analysis, meta-regression).	7
	13f	Describe any sensitivity analyses conducted to assess robustness of the synthesized results.	N/A
Reporting bias assessment	14	Describe any methods used to assess risk of bias due to missing results in a synthesis (arising from reporting biases).	N/A
Certainty assessment	15	Describe any methods used to assess certainty (or confidence) in the body of evidence for an outcome.	N/A
**RESULTS**			
Study selection	16a	Describe the results of the search and selection process, from the number of records identified in the search to the number of studies included in the review, ideally using a flow diagram.	8
	16b	Cite studies that might appear to meet the inclusion criteria, but which were excluded, and explain why they were excluded.	8
Study characteristics	17	Cite each included study and present its characteristics.	8, Table 4
Risk of bias in studies	18	Present assessments of risk of bias for each included study.	Supplementary Table 2
Results of individual studies	19	For all outcomes, present, for each study: (a) summary statistics for each group (where appropriate) and (b) an effect estimate and its precision (e.g. confidence/credible interval), ideally using structured tables or plots.	Table 4, Forest plots
Results of syntheses	20a	For each synthesis, briefly summarise the characteristics and risk of bias among contributing studies.	Table 4
	20b	Present results of all statistical syntheses conducted. If meta-analysis was done, present for each the summary estimate and its precision (e.g. confidence/credible	Forest plots

		interval) and measures of statistical heterogeneity. If comparing groups, describe the direction of the effect.	
	20c	Present results of all investigations of possible causes of heterogeneity among study results.	Forest plots, subgroup analyses
	20d	Present results of all sensitivity analyses conducted to assess the robustness of the synthesized results.	N/A
Reporting biases	21	Present assessments of risk of bias due to missing results (arising from reporting biases) for each synthesis assessed.	N/A
Certainty of evidence	22	Present assessments of certainty (or confidence) in the body of evidence for each outcome assessed.	N/A
**DISCUSSION**			
Discussion	23a	Provide a general interpretation of the results in the context of other evidence.	20
	23b	Discuss any limitations of the evidence included in the review.	20,21
	23c	Discuss any limitations of the review processes used.	20,21
	23d	Discuss implications of the results for practice, policy, and future research.	21
**OTHER INFORMATION**			
Registration and protocol	24a	Provide registration information for the review, including register name and registration number, or state that the review was not registered.	N/A
	24b	Indicate where the review protocol can be accessed, or state that a protocol was not prepared.	5,6
	24c	Describe and explain any amendments to information provided at registration or in the protocol.	N/A
Support	25	Describe sources of financial or non-financial support for the review, and the role of the funders or sponsors in the review.	21
Competing interests	26	Declare any competing interests of review authors.	22
Availability of data, code and other materials	27	Report which of the following are publicly available and where they can be found: template data collection forms; data extracted from included studies; data used for all analyses; analytic code; any other materials used in the review.	N/A

Supplementary Table 2. Quality analysis based on Newcastle-Ottawa Scale.

Study	Selection (4)	Comparability (2)	Exposure (3)	NOS Score
Trabace 2002	2	1	2	5
Rainero 2004	4	2	2	8
Herken 2005	3	1	2	6
Mazaheri 2006	3	2	2	7
Lee 2007	3	2	2	7
Ghosh 2009	3	2	2	7
Asuni 2009	3	1	2	6
Schürks 2009	4	1	2	7
Pappa 2010	3	1	2	6
Yılmaz 2010	3	1	2	6
Ates 2011	3	2	2	7
Stuart 2013	4	2	2	8
Fawzi 2015	4	2	2	8
Shaik 2018	2	2	2	6
Hamad 2021	4	1	2	7
Kesavan 2021	4	1	2	7
Tatlisuluoglu 2021	2	2	2	6
Huang 2025	3	2	2	7
Liampas 2026	4	2	2	8

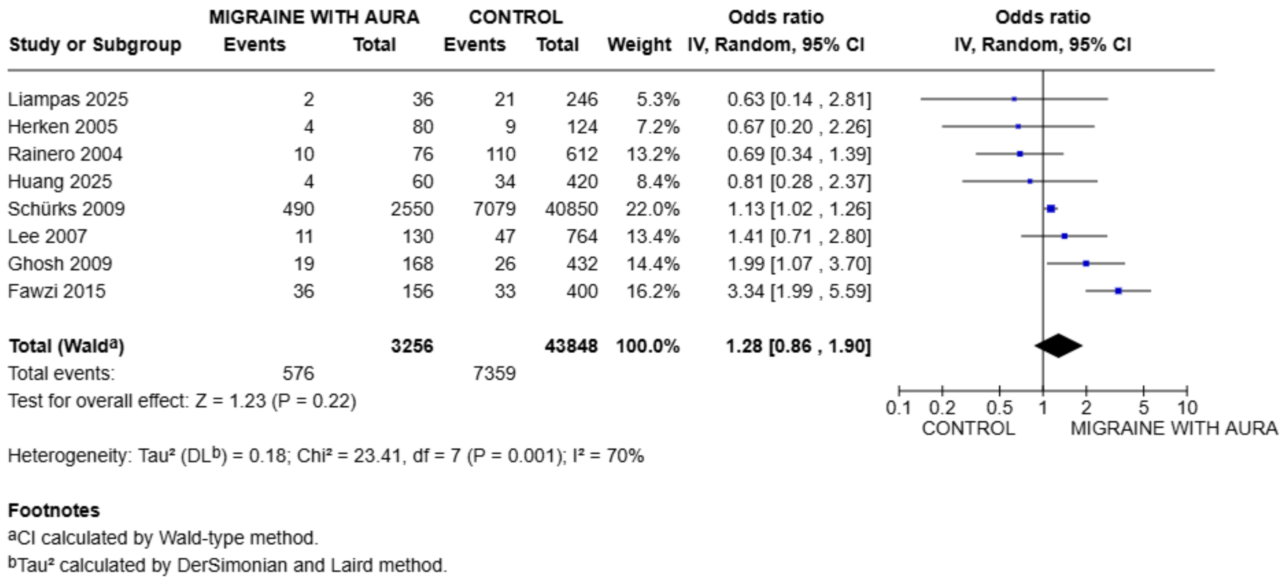

Figure S1. Association between rs1800629 and migraine with aura– allelic model.

Study or Subgroup	MIGRAINE WITHOUT AURA Events	Total	CONTROL Events	Total	Weight	Odds ratio IV, Random, 95% CI
Rainero 2004	34	522	110	612	11.5%	0.32 [0.21 , 0.48]
Asuni 2009	28	598	30	556	10.4%	0.86 [0.51 , 1.46]
Liampas 2025	11	136	21	246	8.3%	0.94 [0.44 , 2.02]
Huang 2025	28	364	34	420	10.4%	0.95 [0.56 , 1.59]
Schürks 2009	662	3902	7079	40850	13.4%	0.97 [0.89 , 1.06]
Herken 2005	3	40	9	124	4.5%	1.04 [0.27 , 4.03]
Lee 2007	46	654	47	764	11.3%	1.15 [0.76 , 1.76]
Ghosh 2009	22	264	26	432	9.8%	1.42 [0.79 , 2.56]
Fawzi 2015	41	244	33	400	10.7%	2.25 [1.38 , 3.66]
Yılmaz 2010	37	134	18	192	9.6%	3.69 [1.99 , 6.82]
**Total (Wald[a])**		**6858**		**44596**	**100.0%**	**1.11 [0.78 , 1.58]**
Total events:	912		7407			

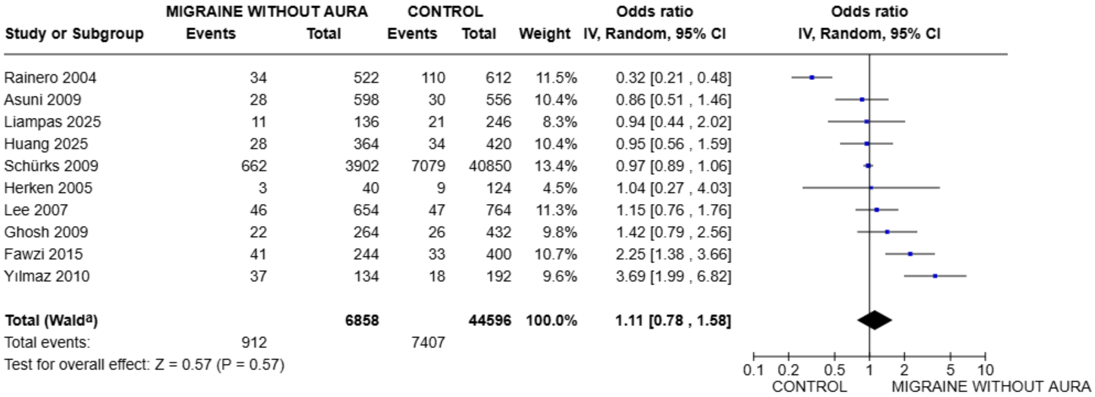

Test for overall effect: Z = 0.57 (P = 0.57)

Heterogeneity: Tau² (DL[b]) = 0.24; Chi² = 60.98, df = 9 (P < 0.00001); I² = 85%

Footnotes

[a]CI calculated by Wald-type method.

[b]Tau² calculated by DerSimonian and Laird method.

Figure S2. Association between rs1800629 and migraine without aura – allelic model.

Study or Subgroup	MIGRAINE Events	Total	CONTROL Events	Total	Weight	Odds ratio IV, Random, 95% CI
Rainero 2004	29	430	80	462	15.3%	0.35 [0.22 , 0.54]
Liampas 2025	17	210	15	152	11.8%	0.80 [0.39 , 1.67]
Asuni 2009	25	522	15	288	12.6%	0.92 [0.47 , 1.77]
Schürks 2009	1623	9154	7079	40850	18.6%	1.03 [0.97 , 1.09]
Lee 2007	63	878	47	764	16.0%	1.18 [0.80 , 1.74]
Ghosh 2009	31	304	19	304	13.4%	1.70 [0.94 , 3.09]
Yılmaz 2010	31	114	14	166	12.3%	4.06 [2.04 , 8.05]
**Total (Wald[a])**		**11612**		**42986**	**100.0%**	**1.08 [0.71 , 1.63]**
Total events:	1819		7269			

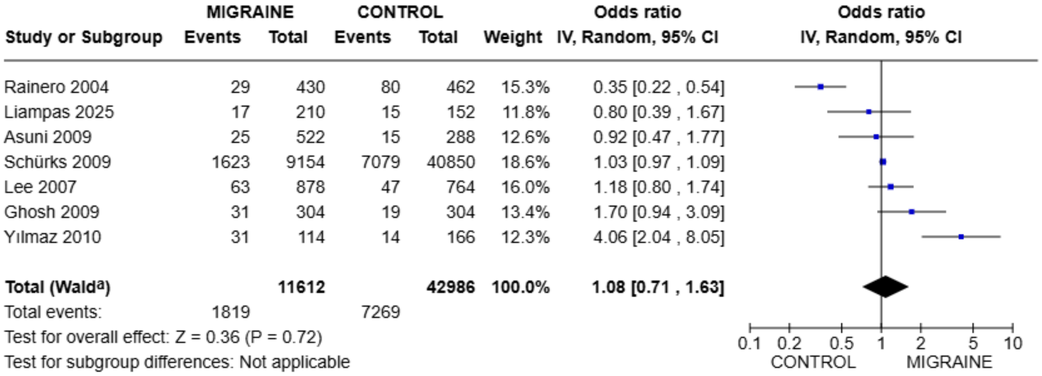

Test for overall effect: Z = 0.36 (P = 0.72)

Test for subgroup differences: Not applicable

Heterogeneity: Tau² (DL[b]) = 0.24; Chi² = 42.03, df = 6 (P < 0.00001); I² = 86%

Footnotes

[a]CI calculated by Wald-type method.

[b]Tau² calculated by DerSimonian and Laird method.

Figure S3. Association between rs1800629 and migraine in women – allelic model.

Study or Subgroup	MIGRAINE Events	Total	CONTROL Events	Total	Weight	Odds ratio IV, Fixed, 95% CI
Rainero 2004	15	168	30	150	47.7%	0.39 [0.20 , 0.76]
Asuni 2009	3	76	15	268	13.1%	0.69 [0.20 , 2.46]
Liampas 2025	2	36	6	94	7.7%	0.86 [0.17 , 4.49]
Ghosh 2009	10	128	7	128	21.1%	1.46 [0.54 , 3.98]
Yılmaz 2010	6	20	4	26	10.3%	2.36 [0.56 , 9.87]
**Total**		**428**		**666**	**100.0%**	**0.71 [0.45 , 1.13]**
Total events:	36		62			

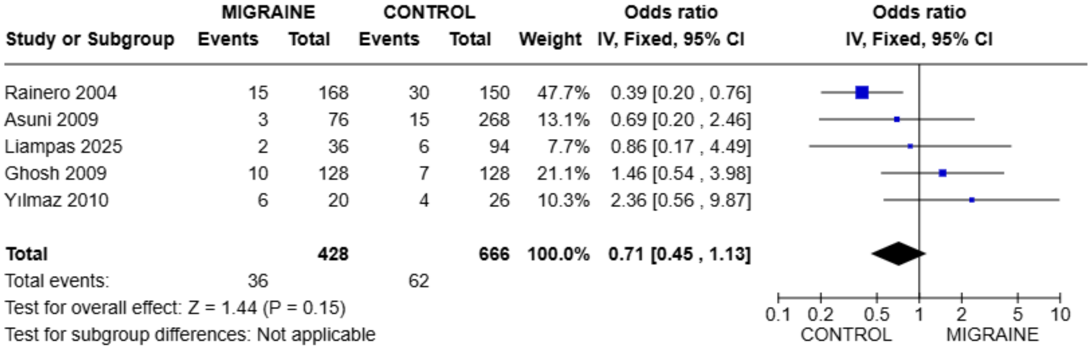

Test for overall effect: Z = 1.44 (P = 0.15)

Test for subgroup differences: Not applicable

Heterogeneity: Chi² = 7.84, df = 4 (P = 0.10); I² = 49%

Figure S4. Association between rs1800629 and migraine in men – allelic model.

Study or Subgroup	MIGRAINE Events	MIGRAINE Total	CONTROL Events	CONTROL Total	Weight	Odds ratio IV, Random, 95% CI
Rainero 2004	44	598	110	612	20.5%	0.36 [0.25 , 0.52]
Liampas 2025	18	246	21	246	14.1%	0.85 [0.44 , 1.63]
Asuni 2009	28	598	30	556	16.8%	0.86 [0.51 , 1.46]
Schürks 2009	1623	9154	7079	40850	25.7%	1.03 [0.97 , 1.09]
Stuart 2013	135	670	133	690	22.8%	1.06 [0.81 , 1.38]
**Total (Wald[a])**		**11266**		**42954**	**100.0%**	**0.79 [0.55 , 1.14]**
Total events:	1848		7373			

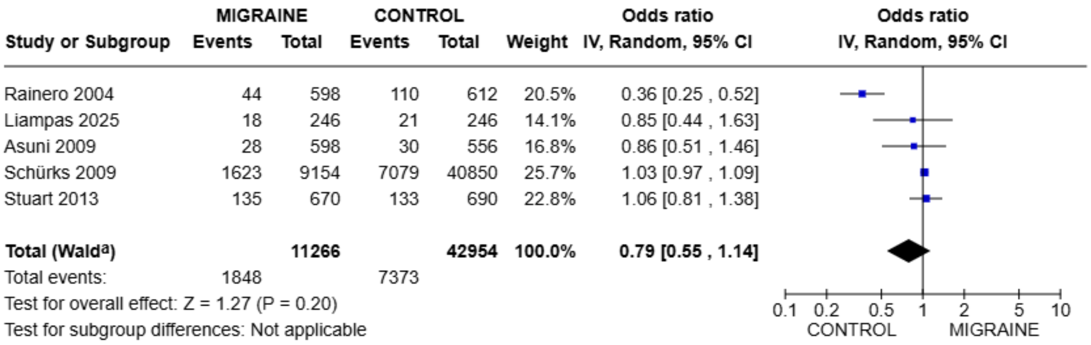

Test for overall effect: Z = 1.27 (P = 0.20)
Test for subgroup differences: Not applicable
Heterogeneity: Tau^2 (DL[b]) = 0.13; Chi^2 = 30.47, df = 4 (P < 0.00001); I^2 = 87%

Footnotes
[a]CI calculated by Wald-type method.
[b]Tau^2 calculated by DerSimonian and Laird method.

Figure S5. Association between rs1800629 and migraine in individuals of Caucasian ancestry – allelic model.

Study or Subgroup	MIGRAINE Events	MIGRAINE Total	CONTROL Events	CONTROL Total	Weight	Odds ratio IV, Random, 95% CI
Ates 2011	0	203	0	202		Not estimable
Shaik 2018	0	129	0	129		Not estimable
Rainero 2004	1	299	11	306	5.5%	0.09 [0.01 , 0.70]
Huang 2025	0	212	2	210	2.8%	0.20 [0.01 , 4.11]
Lee 2007	1	439	3	382	4.7%	0.29 [0.03 , 2.78]
Ghosh 2009	0	216	1	216	2.6%	0.33 [0.01 , 8.19]
Asuni 2009	1	299	1	278	3.3%	0.93 [0.06 , 14.93]
Schürks 2009	127	4577	601	20425	25.5%	0.94 [0.78 , 1.14]
Liampas 2025	1	123	1	123	3.3%	1.00 [0.06 , 16.17]
Stuart 2013	20	335	18	345	19.1%	1.15 [0.60 , 2.22]
Kesavan 2021	16	212	10	218	16.6%	1.70 [0.75 , 3.83]
Herken 2005	1	60	0	62	2.6%	3.15 [0.13 , 78.89]
Fawzi 2015	13	200	2	200	8.7%	6.88 [1.53 , 30.91]
Yılmaz 2010	7	67	1	96	5.3%	11.08 [1.33 , 92.34]
**Total (Wald[a])**		**7371**		**23192**	**100.0%**	**1.17 [0.68 , 2.01]**
Total events:	188		651			

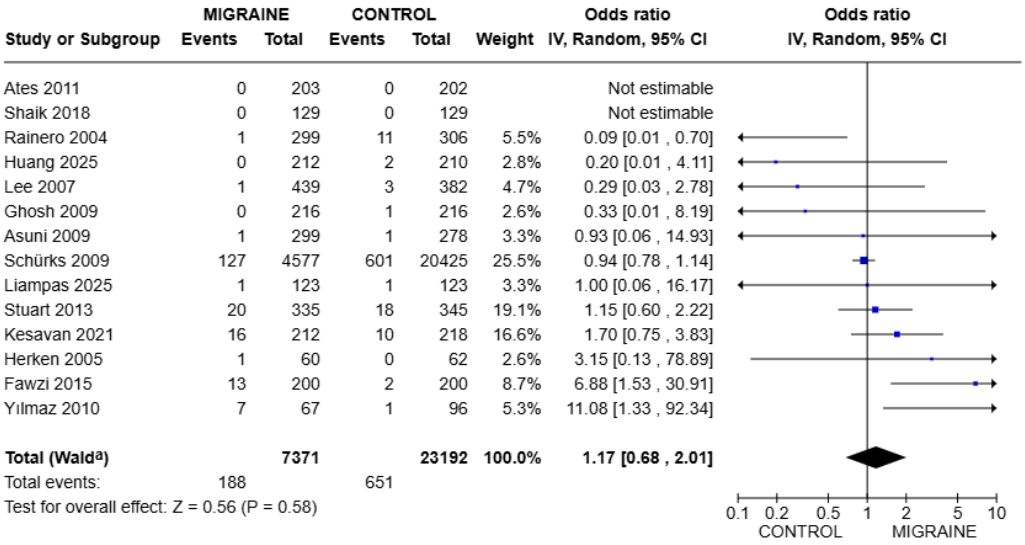

Test for overall effect: Z = 0.56 (P = 0.58)

Heterogeneity: Tau^2 (DL[b]) = 0.29; Chi^2 = 21.94, df = 11 (P = 0.02); I^2 = 50%

Footnotes
[a]CI calculated by Wald-type method.
[b]Tau^2 calculated by DerSimonian and Laird method.

Figure S6. Association between rs1800629 and migraine – recessive model.

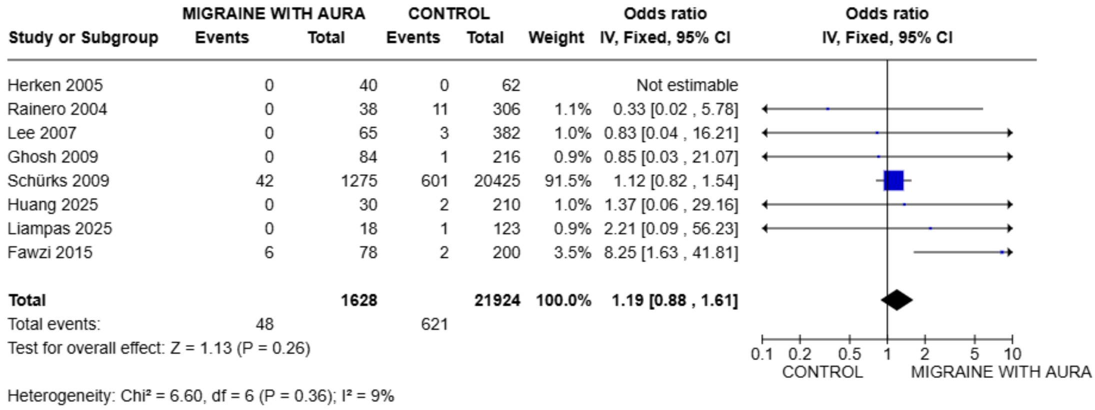

Figure S7. Association between rs1800629 and migraine with aura – recessive model.

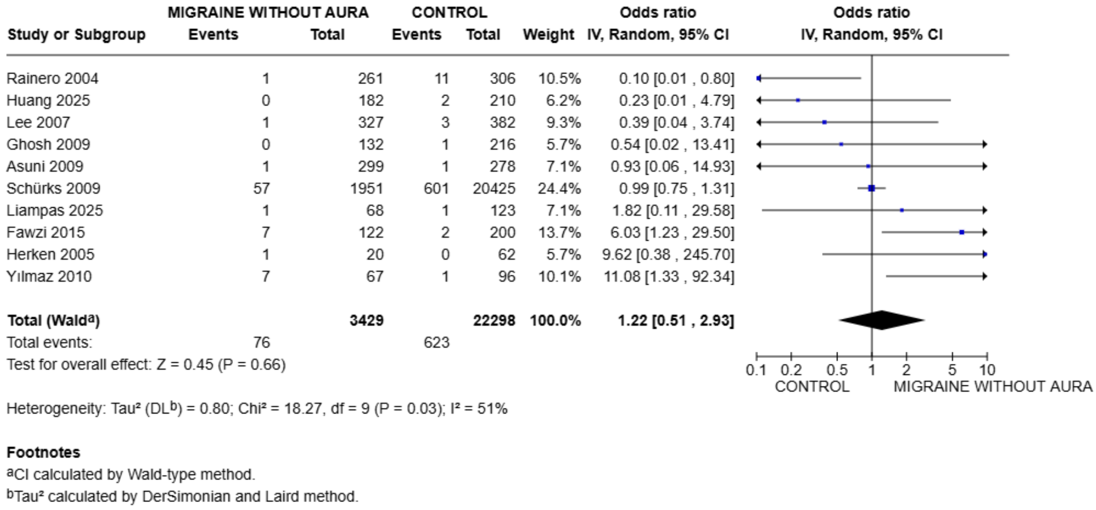

Figure S8. Association between rs1800629 and migraine without aura – recessive model.

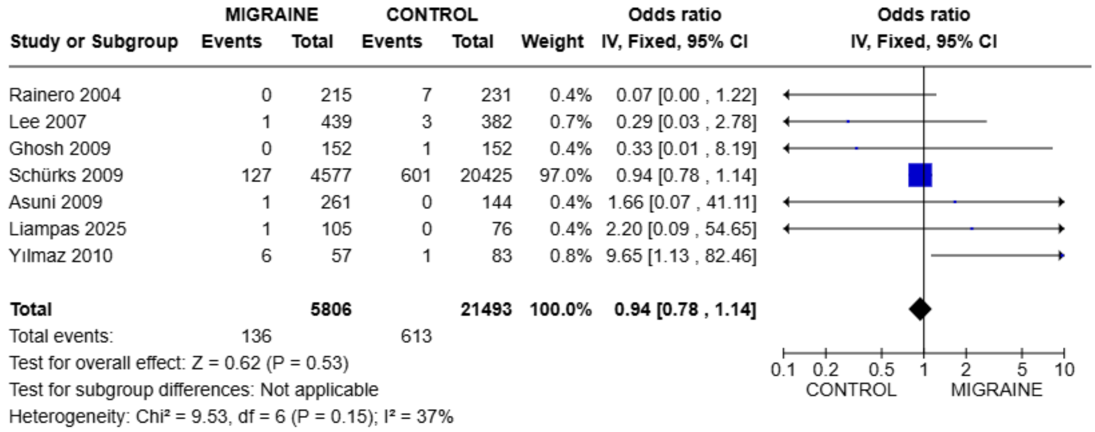

Figure S9. Association between rs1800629 and migraine in women – recessive model.

Study or Subgroup	MIGRAINE Events	MIGRAINE Total	CONTROL Events	CONTROL Total	Weight	Odds ratio IV, Fixed, 95% CI
Ghosh 2009	0	64	0	64		Not estimable
Rainero 2004	1	83	4	75	41.9%	0.22 [0.02 , 1.98]
Liampas 2025	0	18	1	47	19.5%	0.84 [0.03 , 21.51]
Asuni 2009	0	38	1	134	19.8%	1.16 [0.05 , 28.95]
Yılmaz 2010	1	9	0	13	18.7%	4.76 [0.17 , 130.96]
**Total**		**212**		**333**	**100.0%**	**0.70 [0.17 , 2.94]**
Total events:	2		6			

Test for overall effect: Z = 0.49 (P = 0.63)
Test for subgroup differences: Not applicable
Heterogeneity: Chi² = 2.47, df = 3 (P = 0.48); I² = 0%

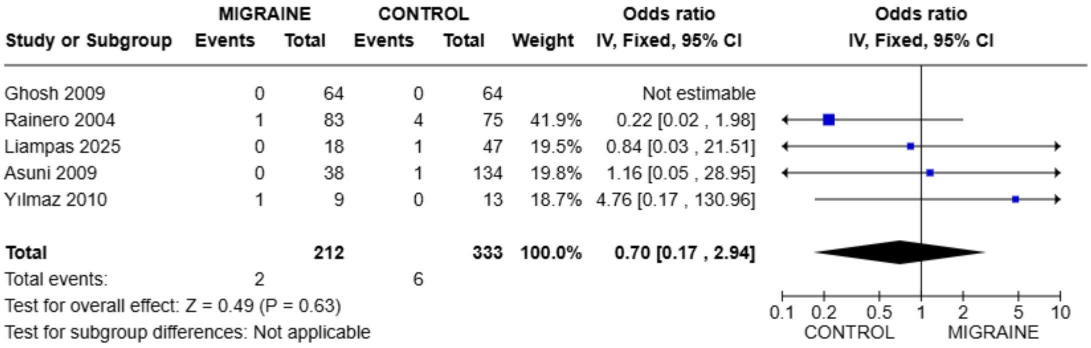

Figure S10. Association between rs1800629 and migraine in men – recessive model.

Study or Subgroup	MIGRAINE Events	MIGRAINE Total	CONTROL Events	CONTROL Total	Weight	Odds ratio M-H, Fixed, 95% CI
Ates 2011	0	203	0	202		Not estimable
Shaik 2018	0	129	0	129		Not estimable
Huang 2025	0	212	2	210	14.3%	0.20 [0.01 , 4.11]
Lee 2007	1	439	3	382	18.3%	0.29 [0.03 , 2.78]
Ghosh 2009	0	216	1	216	8.5%	0.33 [0.01 , 8.19]
Kesavan 2021	16	212	10	218	52.0%	1.70 [0.75 , 3.83]
Herken 2005	1	60	0	62	2.7%	3.15 [0.13 , 78.89]
Yılmaz 2010	7	67	1	96	4.2%	11.08 [1.33 , 92.34]
**Total**		**1538**		**1515**	**100.0%**	**1.54 [0.85 , 2.80]**
Total events:	25		17			

Test for overall effect: Z = 1.42 (P = 0.15)

Heterogeneity: Chi² = 8.31, df = 5 (P = 0.14); I² = 40%

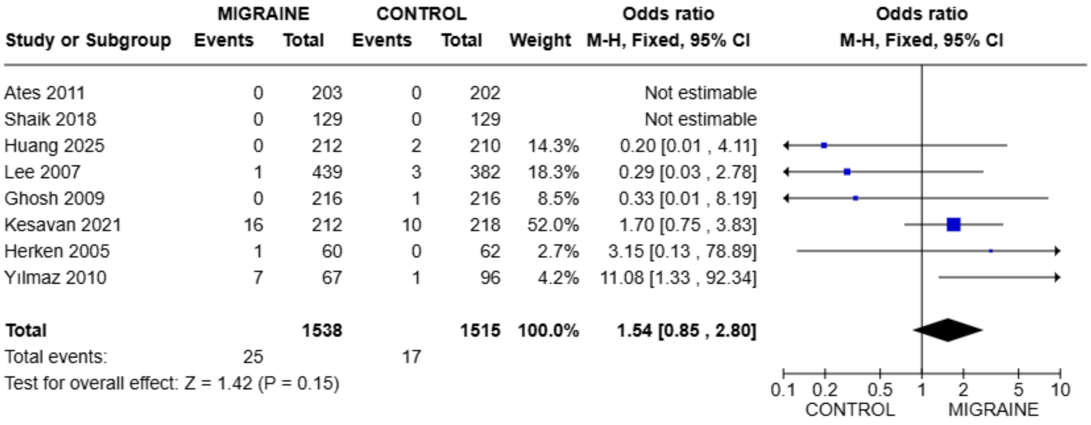

Figure S11. Association between rs1800629 and migraine in individuals of Asian ancestry – recessive model.

Study or Subgroup	MIGRAINE Events	MIGRAINE Total	CONTROL Events	CONTROL Total	Weight	Odds ratio IV, Fixed, 95% CI
Rainero 2004	1	299	11	306	0.8%	0.09 [0.01 , 0.70]
Asuni 2009	1	299	1	278	0.4%	0.93 [0.06 , 14.93]
Schürks 2009	127	4577	601	20425	90.4%	0.94 [0.78 , 1.14]
Liampas 2025	1	123	1	123	0.4%	1.00 [0.06 , 16.17]
Stuart 2013	20	335	18	345	7.9%	1.15 [0.60 , 2.22]
**Total**		**5633**		**21477**	**100.0%**	**0.94 [0.78 , 1.13]**
Total events:	150		632			

Test for overall effect: Z = 0.67 (P = 0.50)
Test for subgroup differences: Not applicable
Heterogeneity: Chi² = 5.39, df = 4 (P = 0.25); I² = 26%

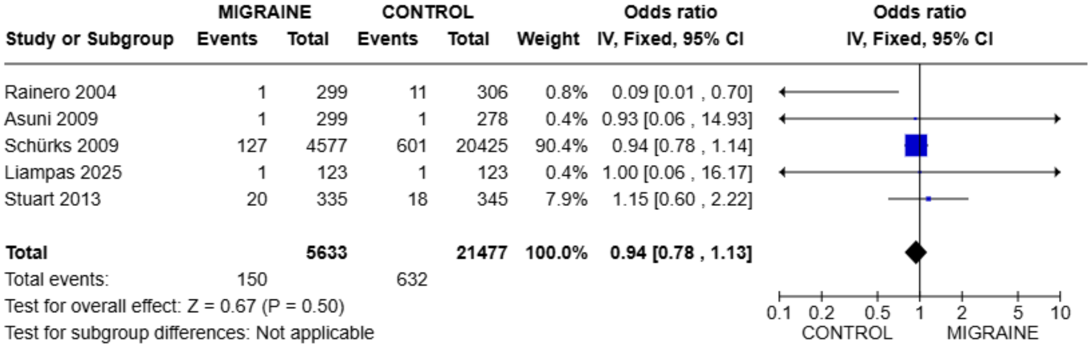

Figure S12. Association between rs1800629 and migraine in individuals of Caucasian ancestry – recessive model.

Study or Subgroup	MIGRAINE Events	MIGRAINE Total	CONTROL Events	CONTROL Total	Weight	Odds ratio IV, Random, 95% CI
Rainero 2004	43	299	99	306	8.2%	0.35 [0.23 , 0.53]
Herken 2005	6	60	9	62	4.0%	0.65 [0.22 , 1.97]
Kesavan 2021	54	212	66	218	8.0%	0.79 [0.52 , 1.20]
Liampas 2025	17	123	20	123	6.1%	0.83 [0.41 , 1.67]
Asuni 2009	27	299	29	278	7.1%	0.85 [0.49 , 1.48]
Huang 2025	32	212	32	210	7.3%	0.99 [0.58 , 1.68]
Schürks 2009	1496	4577	6478	20425	9.7%	1.05 [0.98 , 1.12]
Stuart 2013	115	335	115	345	8.7%	1.05 [0.76 , 1.44]
Lee 2007	62	439	44	382	8.1%	1.26 [0.84 , 1.91]
Ghosh 2009	41	216	25	216	7.2%	1.79 [1.05 , 3.07]
Ates 2011	78	203	40	202	7.9%	2.53 [1.62 , 3.95]
Fawzi 2015	64	200	31	200	7.6%	2.57 [1.58 , 4.17]
Yılmaz 2010	30	67	17	96	6.1%	3.77 [1.85 , 7.68]
Shaik 2018	30	129	4	129	4.1%	9.47 [3.23 , 27.78]
**Total (Wald[a])**		**7371**		**23192**	**100.0%**	**1.27 [0.96 , 1.70]**
Total events:	2095		7009			

Test for overall effect: Z = 1.65 (P = 0.10)

Heterogeneity: Tau² (DL[b]) = 0.22; Chi² = 92.63, df = 13 (P < 0.00001); I² = 86%

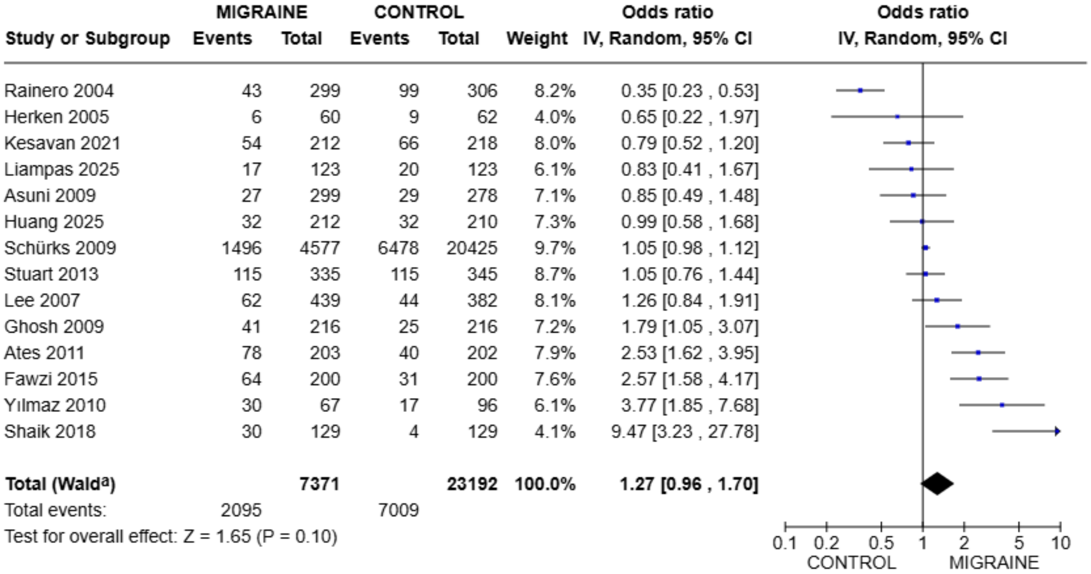

Footnotes
[a]CI calculated by Wald-type method.
[b]Tau² calculated by DerSimonian and Laird method.

Figure S13. Association between rs1800629 and migraine – dominant model.

Study or Subgroup	MIGRAINE WITH AURA Events	MIGRAINE WITH AURA Total	CONTROL Events	CONTROL Total	Weight	Odds ratio IV, Random, 95% CI
Liampas 2025	2	18	20	123	5.1%	0.64 [0.14 , 3.02]
Herken 2005	4	40	9	62	7.0%	0.65 [0.19 , 2.29]
Rainero 2004	10	38	99	306	12.7%	0.75 [0.35 , 1.60]
Huang 2025	4	30	32	210	8.1%	0.86 [0.28 , 2.62]
Schürks 2009	448	1275	6478	20425	23.9%	1.17 [1.04 , 1.31]
Lee 2007	11	65	44	382	13.3%	1.56 [0.76 , 3.22]
Ghosh 2009	19	84	25	216	14.4%	2.23 [1.15 , 4.32]
Fawzi 2015	30	78	31	200	15.6%	3.41 [1.88 , 6.18]
**Total (Wald[a])**		**1628**		**21924**	**100.0%**	**1.35 [0.91 , 2.00]**
Total events:	528		6738			

Test for overall effect: Z = 1.51 (P = 0.13)

Heterogeneity: Tau² (DL[b]) = 0.16; Chi² = 19.25, df = 7 (P = 0.007); I² = 64%

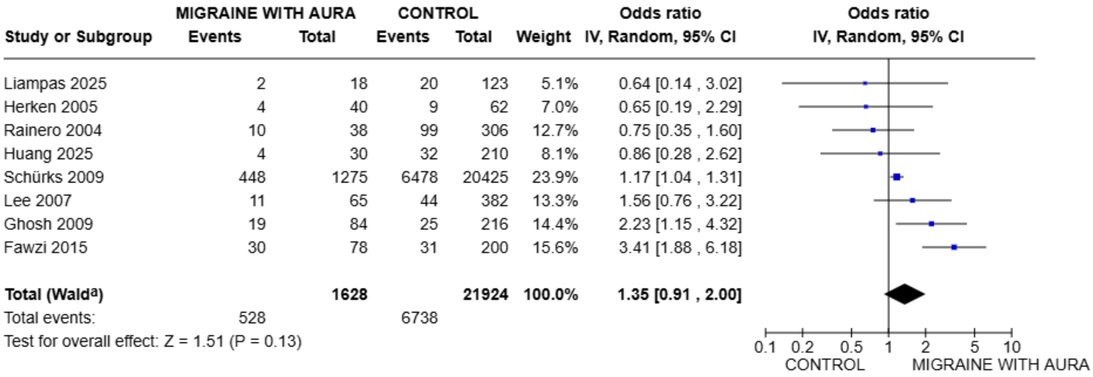

Footnotes
[a]CI calculated by Wald-type method.
[b]Tau² calculated by DerSimonian and Laird method.

Figure S14. Association between rs1800629 and migraine with aura – dominant model.

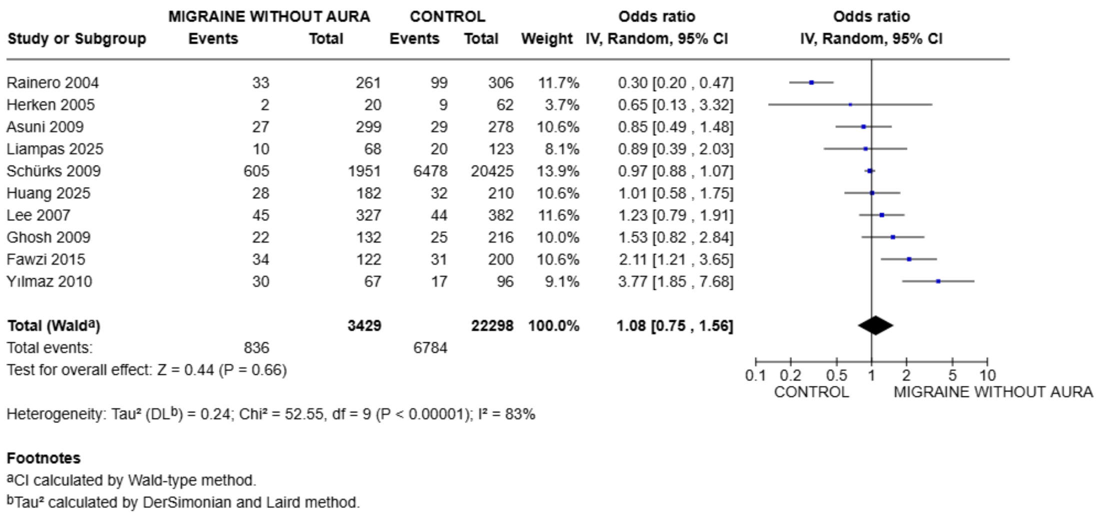

Figure S15. Association between rs1800629 and migraine without aura – dominant model.

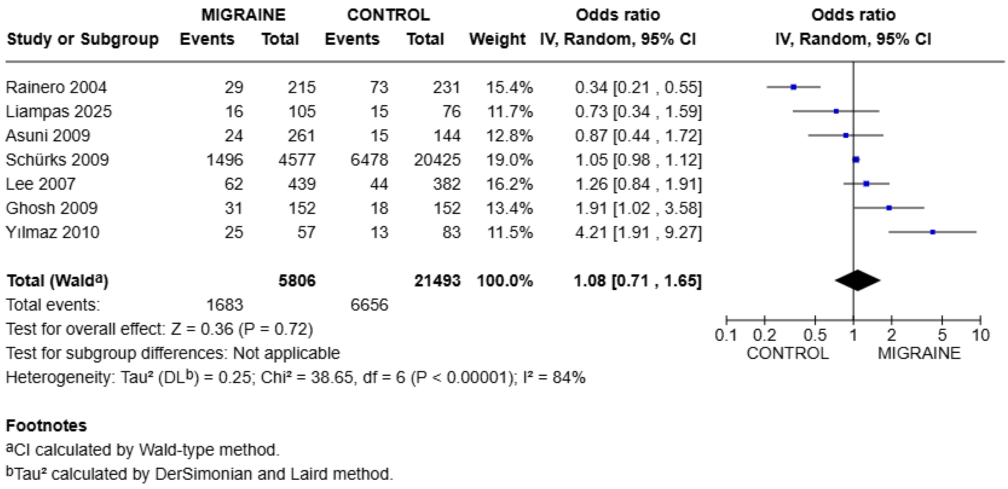

Figure S16. Association between rs1800629 and migraine in women – dominant model.

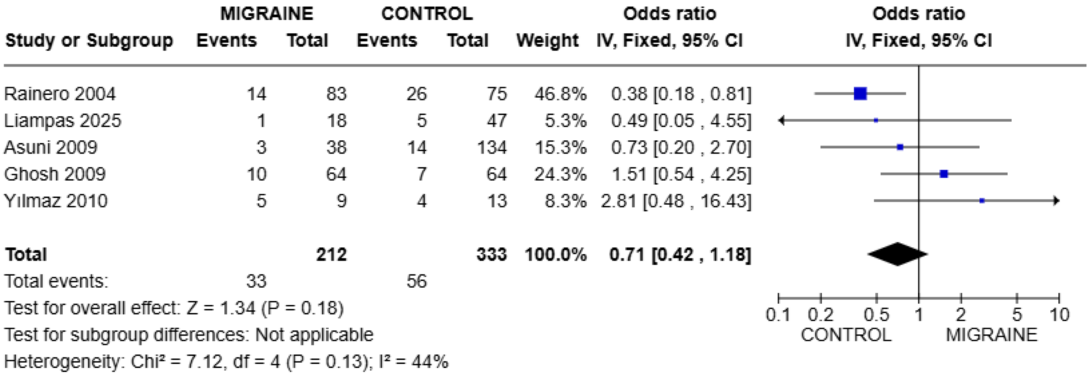

Figure S17. Association between rs1800629 and migraine in men – dominant model.

Study or Subgroup	MIGRAINE Events	MIGRAINE Total	CONTROL Events	CONTROL Total	Weight	Odds ratio IV, Random, 95% CI
Herken 2005	6	60	9	62	8.7%	0.65 [0.22 , 1.97]
Kesavan 2021	54	212	66	218	14.5%	0.79 [0.52 , 1.20]
Huang 2025	32	212	32	210	13.6%	0.99 [0.58 , 1.68]
Lee 2007	62	439	44	382	14.6%	1.26 [0.84 , 1.91]
Ghosh 2009	41	216	25	216	13.5%	1.79 [1.05 , 3.07]
Ates 2011	78	203	40	202	14.3%	2.53 [1.62 , 3.95]
Yılmaz 2010	30	67	17	96	11.9%	3.77 [1.85 , 7.68]
Shaik 2018	30	129	4	129	8.8%	9.47 [3.23 , 27.78]
**Total (Wald[a])**		**1538**		**1515**	**100.0%**	**1.70 [1.06 , 2.72]**
Total events:	333		237			

Test for overall effect: Z = 2.21 (P = 0.03)

Heterogeneity: Tau² (DL[b]) = 0.35; Chi² = 37.47, df = 7 (P < 0.00001); I² = 81%

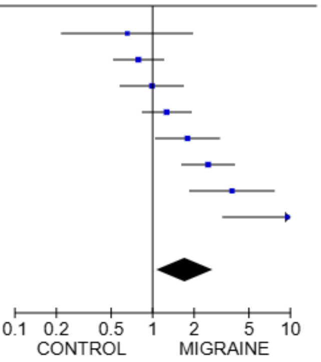

Footnotes
[a]CI calculated by Wald-type method.
[b]Tau² calculated by DerSimonian and Laird method.

Figure S18. Association between rs1800629 and migraine in individuals of Asian ancestry – dominant model.

Study or Subgroup	MIGRAINE Events	MIGRAINE Total	CONTROL Events	CONTROL Total	Weight	Odds ratio IV, Random, 95% CI
Rainero 2004	43	299	99	306	20.5%	0.35 [0.23 , 0.53]
Liampas 2025	17	123	20	123	14.3%	0.83 [0.41 , 1.67]
Asuni 2009	27	299	29	278	17.2%	0.85 [0.49 , 1.48]
Schürks 2009	1496	4577	6478	20425	25.8%	1.05 [0.98 , 1.12]
Stuart 2013	115	335	115	345	22.2%	1.05 [0.76 , 1.44]
**Total (Wald[a])**		**5633**		**21477**	**100.0%**	**0.78 [0.53 , 1.16]**
Total events:	1698		6741			

Test for overall effect: Z = 1.23 (P = 0.22)
Test for subgroup differences: Not applicable
Heterogeneity: Tau² (DL[b]) = 0.16; Chi² = 28.21, df = 4 (P < 0.0001); I² = 86%

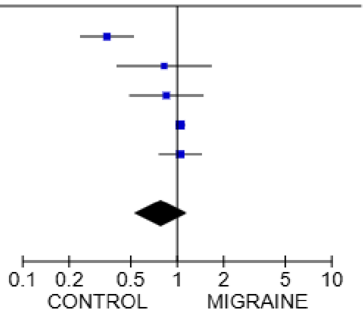

Footnotes
[a]CI calculated by Wald-type method.
[b]Tau² calculated by DerSimonian and Laird method.

Figure S19. Association between rs1800629 and migraine in individuals of Caucasian ancestry – dominant model.

Study or Subgroup	MIGRAINE Events	MIGRAINE Total	CONTROL Events	CONTROL Total	Weight	Odds ratio IV, Random, 95% CI
Rainero 2004	42	299	88	306	8.3%	0.40 [0.27 , 0.61]
Herken 2005	5	60	9	62	3.6%	0.54 [0.17 , 1.70]
Kesavan 2021	38	212	56	218	7.9%	0.63 [0.40 , 1.00]
Liampas 2025	16	123	19	123	6.0%	0.82 [0.40 , 1.68]
Asuni 2009	26	299	28	278	7.1%	0.85 [0.49 , 1.49]
Stuart 2013	95	335	97	345	8.9%	1.01 [0.72 , 1.41]
Schürks 2009	1369	4577	5877	20425	10.2%	1.06 [0.98 , 1.13]
Huang 2025	32	212	30	210	7.3%	1.07 [0.62 , 1.83]
Lee 2007	61	439	41	382	8.2%	1.34 [0.88 , 2.05]
Ghosh 2009	41	216	24	216	7.3%	1.87 [1.09 , 3.23]
Fawzi 2015	51	200	29	200	7.6%	2.02 [1.22 , 3.35]
Ates 2011	78	203	40	202	8.0%	2.53 [1.62 , 3.95]
Yılmaz 2010	23	67	16	96	5.8%	2.61 [1.25 , 5.46]
Shaik 2018	30	129	4	129	3.9%	9.47 [3.23 , 27.78]
**Total (Wald[a])**		**7371**		**23192**	**100.0%**	**1.22 [0.93 , 1.59]**
Total events:	1907		6358			

Test for overall effect: Z = 1.41 (P = 0.16)

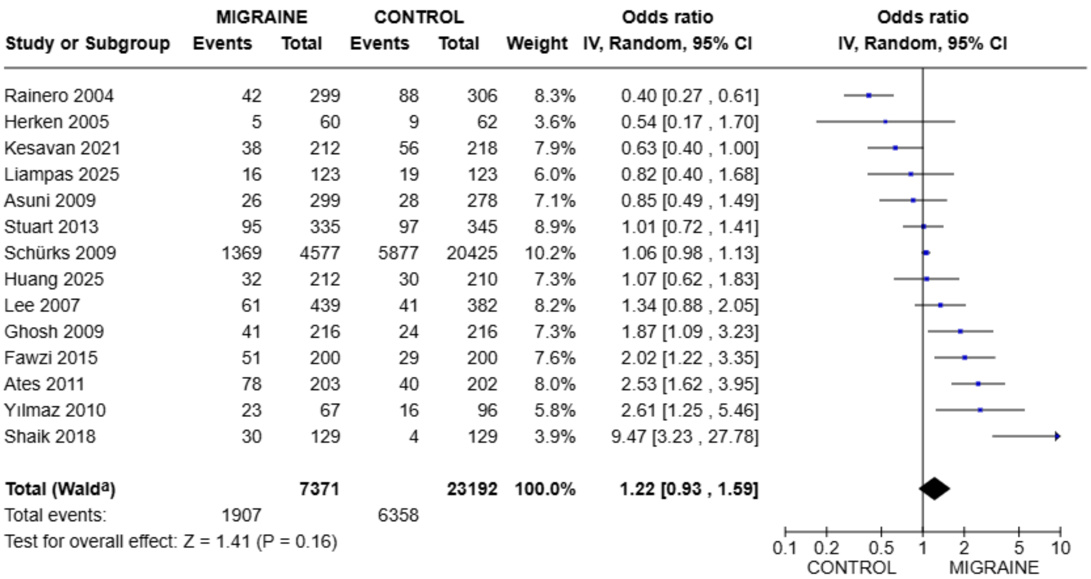

Heterogeneity: Tau^2 (DL[b]) = 0.19; Chi^2 = 76.24, df = 13 (P < 0.00001); I^2 = 83%

Footnotes
[a]CI calculated by Wald-type method.
[b]Tau^2 calculated by DerSimonian and Laird method.

Figure S20. Association between rs1800629 and migraine – over-dominant model.

Study or Subgroup	MIGRAINE WITHOUT AURA Events	MIGRAINE WITHOUT AURA Total	CONTROL Events	CONTROL Total	Weight	Odds ratio IV, Random, 95% CI
Herken 2005	1	20	9	62	1.9%	0.31 [0.04 , 2.61]
Rainero 2004	32	261	88	306	12.2%	0.35 [0.22 , 0.54]
Liampas 2025	9	68	19	123	7.4%	0.83 [0.36 , 1.96]
Asuni 2009	26	299	28	278	10.7%	0.85 [0.49 , 1.49]
Schürks 2009	548	1951	5877	20425	15.9%	0.97 [0.87 , 1.07]
Huang 2025	28	182	30	210	10.7%	1.09 [0.62 , 1.91]
Lee 2007	44	327	41	382	12.1%	1.29 [0.82 , 2.04]
Ghosh 2009	22	132	24	216	9.9%	1.60 [0.86 , 2.99]
Fawzi 2015	27	122	29	200	10.5%	1.68 [0.94 , 3.00]
Yılmaz 2010	23	67	16	96	8.6%	2.61 [1.25 , 5.46]
**Total (Wald[a])**		**3429**		**22298**	**100.0%**	**1.04 [0.75 , 1.42]**
Total events:	760		6161			

Test for overall effect: Z = 0.22 (P = 0.83)

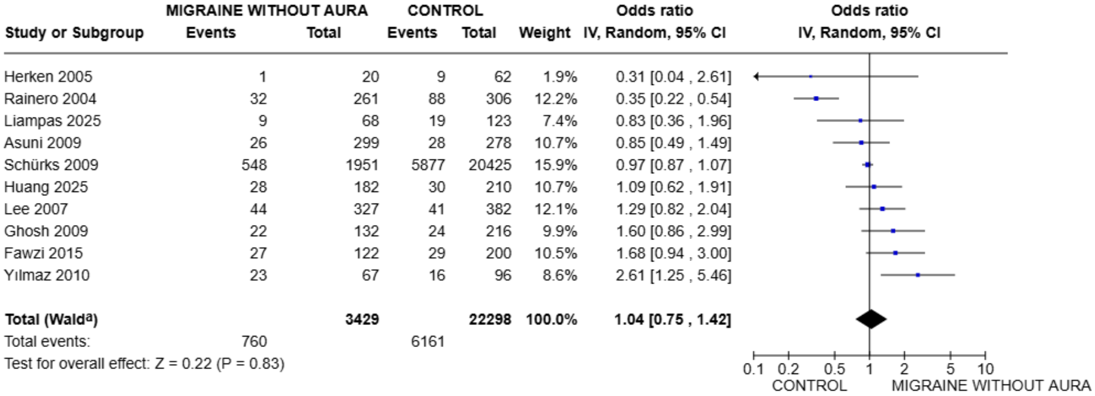

Heterogeneity: Tau^2 (DL[b]) = 0.16; Chi^2 = 36.56, df = 9 (P < 0.0001); I^2 = 75%

Footnotes
[a]CI calculated by Wald-type method.
[b]Tau^2 calculated by DerSimonian and Laird method.

Figure S21. Association between rs1800629 and migraine without aura – over-dominant model.

Study or Subgroup	MIGRAINE Events	MIGRAINE Total	CONTROL Events	CONTROL Total	Weight	Odds ratio IV, Random, 95% CI
Rainero 2004	29	215	66	231	15.6%	0.39 [0.24 , 0.63]
Liampas 2025	15	105	15	76	11.0%	0.68 [0.31 , 1.49]
Asuni 2009	23	261	15	144	12.4%	0.83 [0.42 , 1.65]
Schürks 2009	1369	4577	5877	20425	20.7%	1.06 [0.98 , 1.13]
Lee 2007	61	439	41	382	16.6%	1.34 [0.88 , 2.05]
Ghosh 2009	31	152	17	152	13.1%	2.03 [1.07 , 3.86]
Yılmaz 2010	19	57	12	83	10.6%	2.96 [1.30 , 6.74]
**Total (Wald[a])**		**5806**		**21493**	**100.0%**	**1.06 [0.72 , 1.55]**
Total events:	1547		6043			

Test for overall effect: Z = 0.28 (P = 0.78)
Test for subgroup differences: Not applicable
Heterogeneity: Tau^2 (DL[b]) = 0.18; Chi^2 = 29.23, df = 6 (P < 0.0001); I^2 = 79%

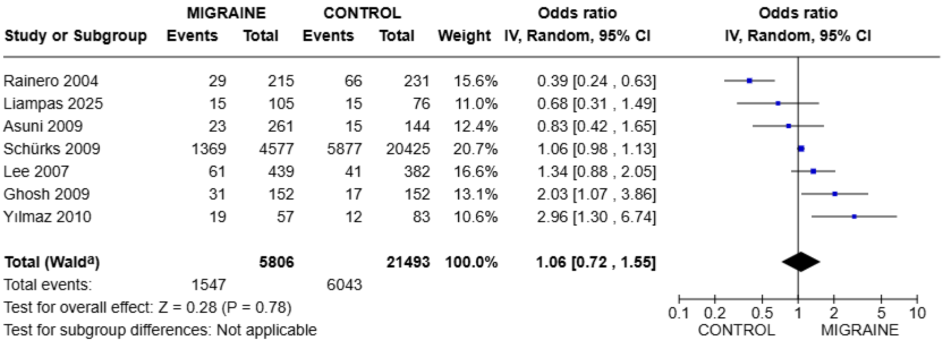

Footnotes
[a]CI calculated by Wald-type method.
[b]Tau^2 calculated by DerSimonian and Laird method.

Figure S22. Association between rs1800629 and migraine in women – over-dominant model.

Study or Subgroup	MIGRAINE Events	MIGRAINE Total	CONTROL Events	CONTROL Total	Weight	Odds ratio IV, Fixed, 95% CI
Rainero 2004	13	83	22	75	45.2%	0.45 [0.21 , 0.97]
Liampas 2025	1	18	4	47	5.3%	0.63 [0.07 , 6.07]
Asuni 2009	3	38	13	134	15.7%	0.80 [0.22 , 2.96]
Ghosh 2009	10	64	7	64	25.2%	1.51 [0.54 , 4.25]
Yılmaz 2010	4	9	4	13	8.7%	1.80 [0.31 , 10.52]
**Total**		**212**		**333**	**100.0%**	**0.76 [0.45 , 1.29]**
Total events:	31		50			

Test for overall effect: Z = 1.01 (P = 0.31)
Test for subgroup differences: Not applicable
Heterogeneity: Chi^2 = 4.44, df = 4 (P = 0.35); I^2 = 10%

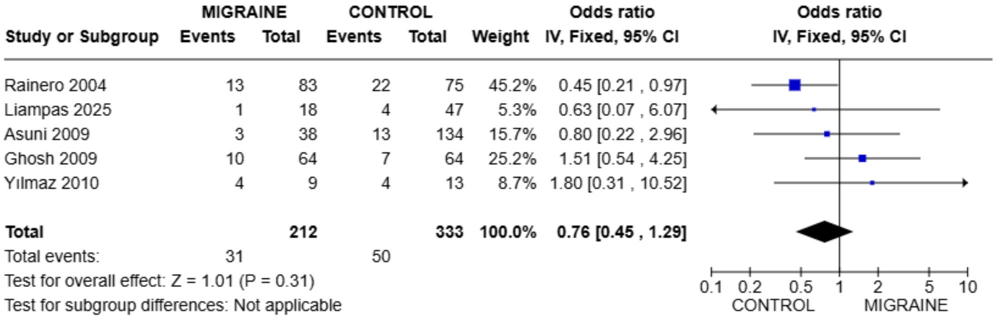

Figure S23. Association between rs1800629 and migraine in men– over-dominant model.

Study or Subgroup	MIGRAINE Events	MIGRAINE Total	CONTROL Events	CONTROL Total	Weight	Odds ratio IV, Random, 95% CI
Herken 2005	5	60	9	62	8.5%	0.54 [0.17 , 1.70]
Kesavan 2021	38	212	56	218	14.3%	0.63 [0.40 , 1.00]
Huang 2025	32	212	30	210	13.6%	1.07 [0.62 , 1.83]
Lee 2007	61	439	41	382	14.6%	1.34 [0.88 , 2.05]
Ghosh 2009	41	216	24	216	13.6%	1.87 [1.09 , 3.23]
Ates 2011	78	203	40	202	14.4%	2.53 [1.62 , 3.95]
Yılmaz 2010	23	67	16	96	11.9%	2.61 [1.25 , 5.46]
Shaik 2018	30	129	4	129	9.1%	9.47 [3.23 , 27.78]
**Total (Wald[a])**		**1538**		**1515**	**100.0%**	**1.60 [0.99 , 2.60]**
Total events:	308		220			

Test for overall effect: Z = 1.91 (P = 0.06)

Heterogeneity: Tau^2 (DL[b]) = 0.37; Chi^2 = 37.45, df = 7 (P < 0.00001); I^2 = 81%

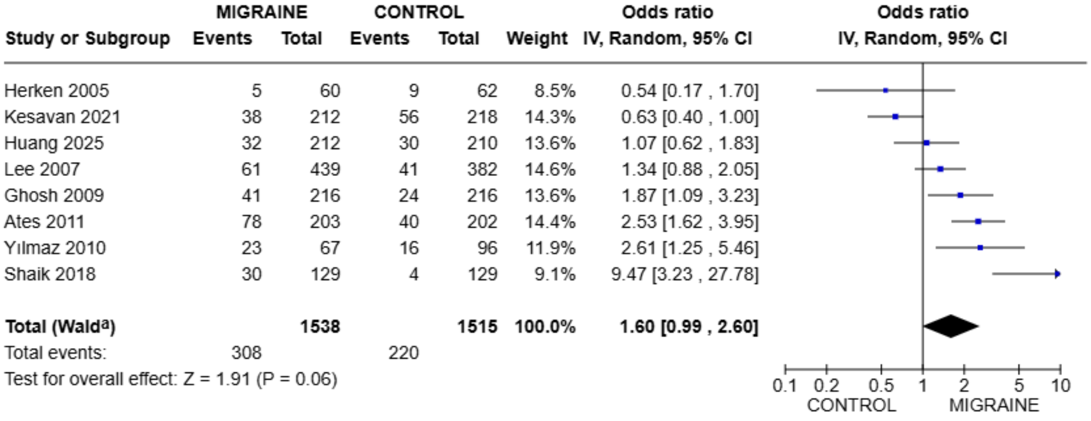

Footnotes
[a]CI calculated by Wald-type method.
[b]Tau^2 calculated by DerSimonian and Laird method.

Figure S24. Association between rs1800629 and migraine in individuals of Asian ancestry – over-dominant model.

Study or Subgroup	MIGRAINE Events	MIGRAINE Total	CONTROL Events	CONTROL Total	Weight	Odds ratio IV, Random, 95% CI
Rainero 2004	42	299	88	306	20.4%	0.40 [0.27 , 0.61]
Liampas 2025	16	123	19	123	13.1%	0.82 [0.40 , 1.68]
Asuni 2009	26	299	28	278	16.5%	0.85 [0.49 , 1.49]
Stuart 2013	95	335	97	345	22.4%	1.01 [0.72 , 1.41]
Schürks 2009	1369	4577	5877	20425	27.6%	1.06 [0.98 , 1.13]
**Total (Wald[a])**		**5633**		**21477**	**100.0%**	**0.80 [0.56 , 1.15]**
Total events:	1548		6109			

Test for overall effect: Z = 1.21 (P = 0.23)
Test for subgroup differences: Not applicable
Heterogeneity: Tau^2 (DL[b]) = 0.12; Chi^2 = 21.28, df = 4 (P = 0.0003); I^2 = 81%

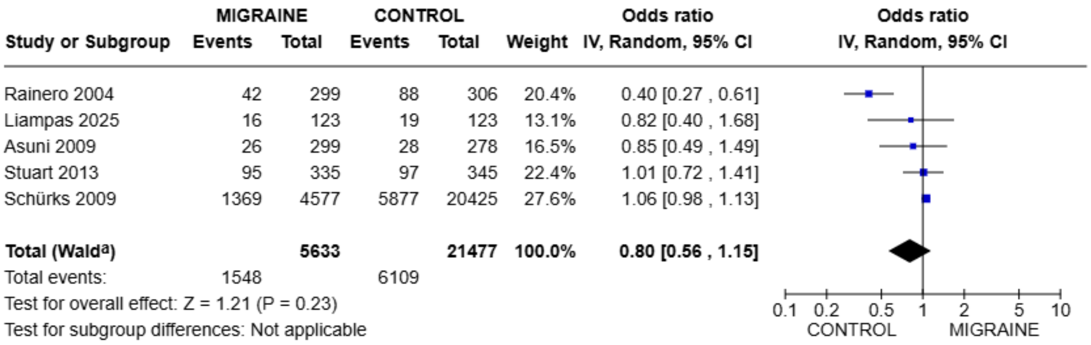

Footnotes
[a]CI calculated by Wald-type method.
[b]Tau^2 calculated by DerSimonian and Laird method.

Figure S25. Association between rs1800629 and migraine in individuals of Caucasian ancestry – over-dominant model.

AIMS Press

© 2026 the Author(s), licensee AIMS Press. This is an open access article distributed under the terms of the Creative Commons Attribution License (https://creativecommons.org/licenses/by/4.0)
